# Imaging Metabolic Flow of Water in Plants with Isotope‐Traced Stimulated Raman Scattering Microscopy

**DOI:** 10.1002/advs.202407543

**Published:** 2024-09-20

**Authors:** Simin Bi, Jianpeng Ao, Ting Jiang, Xianmiao Zhu, Yimin Zhu, Weibing Yang, Binglian Zheng, Minbiao Ji

**Affiliations:** ^1^ State Key Laboratory of Surface Physics and Department of Physics Academy for Engineering and Technology Human Phenome Institute Key Laboratory of Micro and Nano Photonic Structures (Ministry of Education) Shanghai Key Laboratory of Metasurfaces for Light Manipulation Fudan University Shanghai 200433 China; ^2^ State Key Laboratory of Genetic Engineering Institute of Plant Biology School of Life Sciences Fudan University Shanghai 200438 China; ^3^ National Key Laboratory of Plant Molecular Genetics CAS Center for Excellence in Molecular Plant Sciences Institute of Plant Physiology and Ecology Chinese Academy of Sciences Shanghai 200032 China; ^4^ CAS‐JIC Center of Excellence for Plant and Microbial Sciences (CEPAMS) Institute of Plant Physiology and Ecology Chinese Academy of Sciences Shanghai 200032 China

**Keywords:** label‐free imaging, plant science, stimulated Raman scattering, water metabolism

## Abstract

Water plays a vital role in the life cycle of plants, participating in various critical biochemical reactions during both non‐photosynthetic and photosynthetic processes. Direct visualization of the metabolic activities of water in plants with high spatiotemporal resolution is essential to reveal the functional utilization of water. Here, stimulated Raman scattering (SRS) microscopy is applied to monitor the metabolic processes of deuterated water (D_2_O) in model plant *Arabidopsis thaliana (A. thaliana)*. The work shows that in plants uptaking D_2_O/water solution, proton‐transfer from water to organic metabolites results in the formation of C‐D bonds in newly synthesized biomolecules (lipid, protein, and polysaccharides, etc.) that allow high‐resolution detection with SRS. Reversible metabolic pathways of oil‐starch conversion between seed germination and seed development processes are verified. Spatial heterogeneity of metabolic activities along the vertical axis of plants (root, stem, and tip meristem), as well as the radial distributions of secondary growth on the horizontal cross‐sections are quantified. Furthermore, metabolic flow of protons from plants to animals is visualized in aphids feeding on *A. thaliana*. Collectively, SRS microscopy has potential to trace a broad range of matter flows in plants, such as carbon storage and nutrition metabolism.

## Graphical abstract

High‐resolution optical imaging of water metabolism in plant is crucial for studying the functional activity of water, including photosynthesis and numerous biochemical reactions. Here, stimulated Raman scattering (SRS) microscopy integrated with isotope tracing of deuterated water allows the visualization of metabolic dynamics of water in the whole plant with subcellular resolution, and throughout the lifespan of plant.

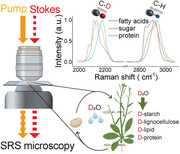

## 1 Introduction

Essential for life, water plays a crucial role in plant physiology and biochemistry. Water constitutes 80–95% of mass in a typical growing plant, and is transported throughout the body via diffusion, bulk flow, and osmosis, forming the environment for most biochemical reactions. More importantly, it directly participates in many essential biochemical reactions, including non‐photosynthetic processes at seed germination stage^[^ ^1^ ^]^ and photosynthesis at the growth and developmental stages.^[^ ^2^ ^]^ From the matter flow point‐of‐view, water provides all the hydrogen sources in photosynthesis which further transfer into most of the downstream organic metabolites. Despite the current understanding of many specific biochemical processes at the molecular level, direct visualization of the metabolic activities of water with high spatiotemporal resolution remains technically challenging. However, it is especially important to understand their subcellular/cellular behaviors among various organs and trace the dynamics with physiological changes.

Various techniques have been employed to investigate the metabolic activities in plants. Mass spectrometry (MS) and MS imaging (MSI) are the most widely used technologies for plant researches,^[^ ^3^ ^]^ particularly powerful in proteome analysis and probing specific metabolites including glucosinolates, polyamines, phenolic acids, oligosaccharides, etc. Further combined with isotope‐labeling, MSI was able to trace and quantify small metabolites.^[^ ^4^ ^]^ However, the destructive nature and complex matrices processing have restricted their use for whole living systems. Nuclear magnetic resonance (NMR)^[^ ^5^ ^]^ and positron emission tomography (PET)^[^ ^6^ ^]^ provide noninvasive imaging of metabolic information, but these methods are limited by insufficient spatial resolution (≈100 µm) for cellular/subcellular imaging. Micro autoradiography (MAR) is one of the oldest techniques to measure single‐cell activity with high sensitivity, but it is limited by the use of radio‐active substrates.^[^ ^7^ ^]^ Fluorescence microscopy has been widely adapted in biological and metabolic imaging with remarkable targeting specificity.^[^ ^8^ ^]^ However, the relative bulky fluorophores could not be used to label small water molecules, hence it is difficult to directly probe the metabolic activities of water with fluorescence microscopy. The advantages and disadvantages of current techniques and their temporal‐spatial resolutions are summarized in Table S1 (Supporting Information).

**Figure 1:**
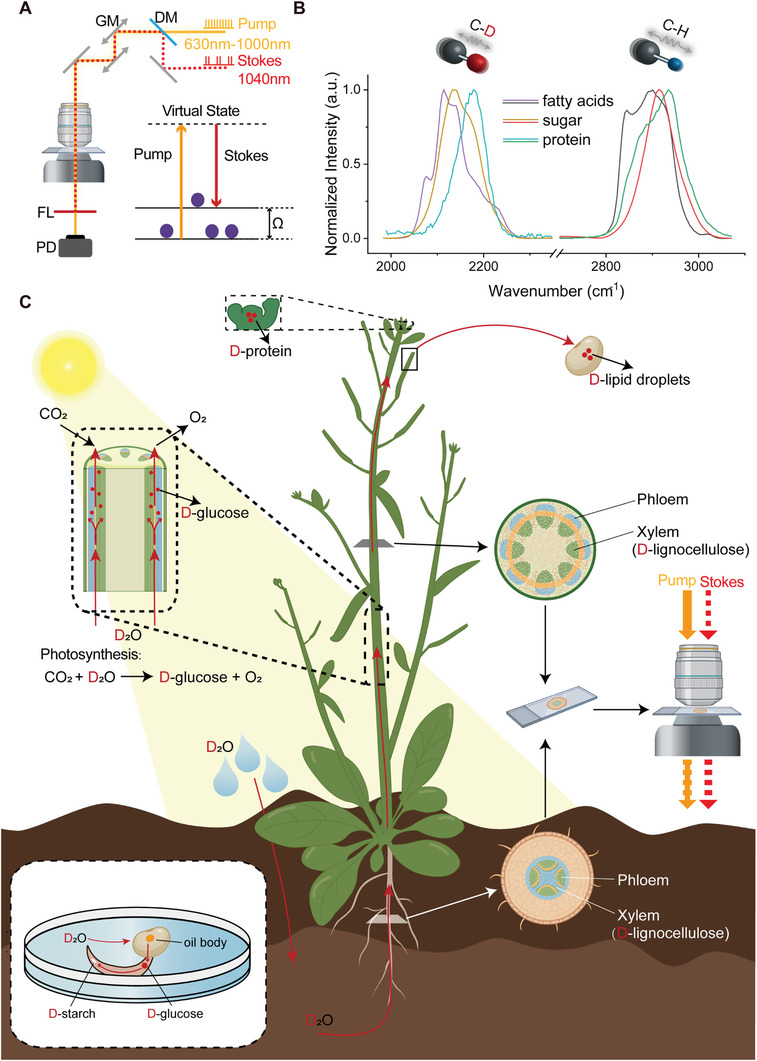
Schematic of experimental approach. A) Principle of SRS microscopy and beam path diagram. DM: dichroic mirror; PD: photodiode; FL: optical filter; GM: galvo mirror. B) C‐D and C‐H bonds produced SRS spectra at distinct peak positions. C‐H spectra were collected from standard samples of oleic acid, glucose and bovine serum albumin. C‐D spectra were collected from deuterated oleic acid, deuterated glucose, and deuterated protein from the abdomen tissue of aphids fed on deuterated *A. thaliana*. C) Illustration of deuterium flow in plants. In seed germination, water participates in the metabolic pathways of oil‐starch conversion (bottom left illustration). In adult plant, water provides most of the hydrogen sources for photosynthesis to generate glucose, which further involves in most metabolic activities in plant. D_2_O transfers deuterium into newly synthesized starch, lipid, protein and lignocellulose, which could be specifically imaged by SRS microscopy.

Raman spectroscopy provides a non‐destructive means to characterize molecular signatures through inelastic scattering between the incoming photons and chemical bond vibrations.^[^ ^9^ ^]^ Native biomolecules are distinguishable based on their intrinsic “fingerprint” Raman spectra, and their isotopologues can be further identified by spectral shifts due to the change of atomic weights.^[^ ^10^ ^]^ Confocal Raman microscopy has been used to image chemical distributions in samples by acquiring Raman spectra pixel‐by‐pixel, but it suffers from relatively slow speed (≈1 ms pixel^−1^) due to weak Raman cross‐section.^[^ ^11^ ^]^ Besides, spontaneous Raman scattering is prone to the interference from autofluorescence background, especially for plant tissues. Coherent Raman scattering microscopy, including coherent anti‐Stokes Raman scattering (CARS) microscopy^[^ ^12^ ^]^ and stimulated Raman scattering (SRS) microscopy^[^ ^13^ ^]^ have been developed as sensitive imaging techniques with high speed (typical ≈2 µs pixel^−1^, could reach ≈60 ns pixel^−1^.^[^ ^14^ ^]^ In particular, SRS amplifies Raman signal (up to ≈10^8^ gain) via stimulated emission process by the mutual interaction of pump and Stokes photons with molecular vibration (**Figure** **1**A). SRS has become a rapid growing chemical imaging tool with major advantages in spectral fidelity, quantitative analysis, insensitive to autofluorescence, and 3D optical sectioning capability (350 nm lateral resolution and 1 µm depth resolution),^[^ ^15^ ^]^ and has been applied to image endogenous molecules in plant tissues, including lignin, polysaccharides, cellulose, etc.^[^ ^15^, ^16^ ^]^

To further enhance the chemical resolution of Raman based techniques, Raman‐tagging and isotope‐labeling have been applied to image small metabolites.^[^ ^17^ ^]^ Compared with fluorescence labels and alkyne‐tags,^[^ ^18^ ^]^ stable isotope labeling introduces minimal disruption and toxicity to biological processes. Deuterium substituted O‐D and C‐D bonds exhibit red‐shifted vibrational frequencies (compared with O‐H and C‐H bonds) in the cell‐silent spectral range (1800‐2700 cm^−1^) with enhanced specificity and reduced background. Spontaneous Raman measurement of deuterated water (D_2_O) has been successfully applied to evaluate water transportation^[^ ^19^ ^]^ and metabolism^[^ ^10^ ^]^ in plant roots. CARS imaging of D_2_O has allowed the monitoring of transient water exchange in live microbial cells.^[^ ^20^ ^]^ More studies have been conducted using SRS microscopy combined with deuterated small molecules on the metabolic processes of various biological systems, including lipid metabolism, protein synthesis, and glucose uptake in microalgae,^[^ ^21^ ^]^ mammalian cells,^[^ ^22^ ^]^ bacteria,^[^ ^23^ ^]^ and animals.^[^ ^24^ ^]^ Despite the previous investigations, demonstration of efficient metabolic imaging of water in the whole plant body is still lacking to reveal the fate of hydrogen/deuterium throughout the lifespan of plant.

In the present work, SRS microscopy combined with isotope tracing was applied to visualize metabolic dynamics of water in model plant *Arabidopsis thaliana (A. thaliana)*. Biosynthesis processes that consume D_2_O and generate C‐D bonds allow the specific detection of newly synthesized biomolecules by their distinct vibrational spectra from the existing C‐H counterpart (Figure 1B). Spatiotemporal evolution and enrichment of C‐D bonds were monitored to demonstrate the spectral tracing of deuterium flow in plants (Figure 1C). Our results verified the switching of metabolic pathways between the germination and development processes of *A. thaliana* seeds. In addition, the secondary growth pattern in the radial cross‐section of roots and stems was visualized, and the metabolic intensity distribution along the axial direction of the stems was quantified to show the spatial profiles of metabolic heterogeneity. Furthermore, the presence of C‐D signal in aphids feeding on *A. thaliana* demonstrated a metabolic flow of deuterium from plants to animals.

## 2 Results

### 2.1 Spectral Tracing of Deuterium Transfer and Metabolic Flow During Seed Germination

First, metabolic activities of water in the seed germination process were investigated. D_2_O‐derived deuterium could be incorporated into C‐D bond through metabolic activities, and the vibrational frequency of C‐D bond is redshifted (compared with C‐H bond) to the cell‐silent spectral region (1800‐2700 cm^−1^) that avoids interference from other endogenous molecules (Figure 1B). To evaluate toxic effect caused by D_2_O in seed germination and plant growth, the germination rates of *A. thaliana* seeds in D_2_O media with different concentrations were analyzed (Figure S1, Supporting Information). As can be seen, higher concentration of D_2_O yielded higher SRS signal of the C‐D bonds, but reduced the germination rate, especial when D_2_O concentration went beyond 50%. Therefore, to balance the germination rate and C‐D signal intensity (Figure S2, Supporting Information), 50% D_2_O/H_2_O was used for most of the subsequent studies, which resulted in moderate change of Arabidopsis growth.^[^ ^25^ ^]^

**Figure 2:**
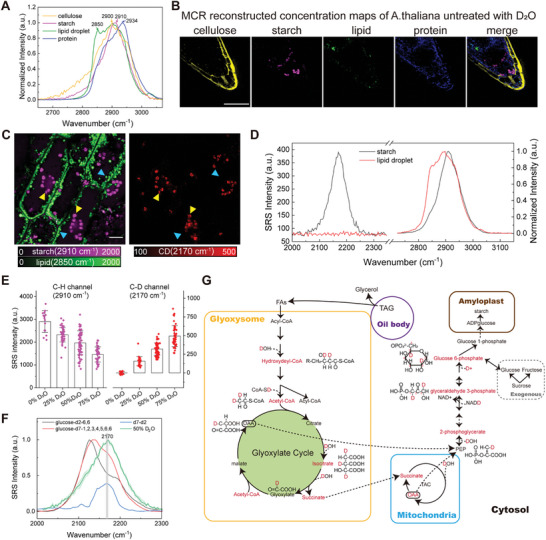
Probing metabolic flow of deuterium from storage oil to starch in the seed germination process. A) Normalized SRS spectra of chemical compositions in seedlings of *A. thaliana* in the C‐H stretch region. B) MCR reconstructed concentration maps of cellulose (yellow), starch (magenta), lipid (green), protein (blue) and their overlay merged image of a seedling of *A. thaliana*, respectively. The images were obtained at C‐H channel. Scale bar, 50 µm. C) Seedlings cultivated with 50% D_2_O/H_2_O show C‐D signal only in starch granules (red), but not in lipid droplets (green), indicating disparate metabolic pathways between lipid (cyan arrowheads) and starch (yellow arrowheads). Left, overlay image of lipid (2850 cm^−1^, green) and starch (2910 cm^−1^, magenta) in C‐H channel obtained by hyperspectral SRS imaging and MCR decomposition. Right, SRS images in C‐D channel (2170 cm^−1^) showing D_2_O‐synthesized starch. Scale bar, 10 µm. D) Mean SRS spectra in the C‐D (left Y‐axes) and normalized SRS spectra in the C‐H stretch range (right Y‐axes) of lipid droplets (cyan arrowheads in (C)) and starch granules (yellow arrowheads in (C)). E) SRS intensities of starch in seedlings in the C‐H and C‐D channels under different D_2_O concentrations. Values are means ± SDs; n = 15 for 0% D_2_O; n = 35 for 25% D_2_O; n = 65 for 50% D_2_O; n = 45 for 75% D_2_O. F) Normalized C‐D spectra of different deuterated glucose and D_2_O‐synthesized starch. The blue curve reflects the difference spectrum between glucose‐d2‐6,6 and glucose‐d7‐1,2,3,4,5,6,6, showing a peak ≈2170 cm^−1^, similar to that of D_2_O‐synthesized starch. G) Proposed deuterium transfer pathways involved in the conversion of storage oil (purple circle) to starch, including lipid β oxidation and glyoxylate cycle in glyoxysome (yellow box), tricarboxylic acid cycle (TAC) in mitochondria (blue box), starch condensation in amyloplast (brown box), exogenous sucrose (dashed box), and gluconeogenesis in the cytosol. TAG, triacylglycerol; FAs, fatty acids; OAA, oxaloacetate; PEP, phosphoenolpyruvate.

*A. thaliana* seeds were treated in 50% D_2_O/H_2_O for ≈7 days (see Methods for details), resulted in the sprouting of radicle (Figure S1A, Supporting Information). Using multivariate curve resolution (MCR) algorithm to analyze hyperspectral SRS images,^[^ ^26^ ^]^ four different chemical constituents (lipid, protein, cellulose, and starch) in C‐H stretch spectral region (**Figure** **2**A, with the SRS spectrum of corresponding standard samples shown in Figure S3, Supporting Information) were differentiated. MCR reconstructed images of a root tip of radicle (Figure 2B) revealed that both lipid and starch were in the form of aggregate droplets/granules. Note that lipid droplets tend to attach to the cell membranes, which often obscured the visualization of cell walls (Figure S4, Supporting Information), as also shown in the 3D imaging of seedling tissue (Movie S1, Supporting Information). By tuning SRS microscopy to target the C‐D stretch vibration (≈2170 cm^−1^), it is found that only starch granules rather than lipid droplets contain C‐D bonds (Figure 2C; Figure S5, Supporting Information). This finding suggests that during the germination of *A. thaliana* seeds, starch granules are synthesized through D_2_O‐participated metabolic processes (C‐D signal indicates newly synthesized molecules), whereas lipids are pre‐stored in the seeds, consistent with the fact that *A. thaliana* seeds are classified as oilseeds.^[^ ^27^ ^]^ The newly generated starch‐rich granules in the radicle tips are statoliths, well‐known for gravity sensing.^[^ ^28^ ^]^ The C‐D spectrum of starch exhibited a typical Raman peak of sugar at 2170 cm^−1^ (Figure 2D). To confirm that the C‐D bonds were indeed originated from enzyme‐catalyzed chemical reactions rather than non‐enzymatic H/D exchanges,^[^ ^24^ ^]^ starch and sucrose were dissolved in D_2_O and rested for ≈7 days, no detectable C‐D signal could be observed in the dry mass (Figure S6, Supporting Information).

Consequently, the amount of newly‐synthesized starch was quantified by analyzing SRS intensities of starch in the C‐D channel at 2170 cm^−1^ under different concentrations of D_2_O. The intensities of C‐D bonds were increased with increasing D_2_O concentration, with the intensities of C‐H bonds exhibited an opposite trend (Figure 2E), both of which showed a roughly linear relationship (Figure S2, Supporting Information). This demonstrates the specific and semi‐quantitative detection of C‐D bonds by SRS microscopy.

To further explore the metabolic process of the deuterium and how it allocates in carbon sites of starches, different deuterium‐labeled glucoses were used in the medium of seeds for spectral tracing (Figure 2F; Figure S7, Supporting Information). Three glucose isotopologues were used: glucose‐d1‐1 with one D1 at the sugar ring which contributes to CD_1_ vibration at 2170–2190 cm^−1^,^[^ ^29^ ^]^ glucose‐d2‐6,6 with two D6's which generates CD_2_ symmetric and anti‐symmetric vibration modes and overtones in Fermi resonance,^[^ ^29^, ^30^ ^]^ and glucose‐d7‐1,2,3,4,5,6,6 with seven D's showing a more complicated spectrum (Figure 2F).^[^ ^30^ ^]^ Subtracting the spectrum of glucose‐d2 from that of glucose‐d7, the remaining signal approximates the contribution resulted from D2‐D5 in sugar rings. The remaining signal displayed a similar peak ≈2170 cm^−1^ to that of starch produced in plants cultivated with D_2_O, implying CD_1_ vibration in sugar rings. Besides, starch Raman spectra from plants separately cultured with the three glucose isotopologues dissolved in pure H_2_O or 50% D_2_O/H_2_O were obtained. Spectra of plant starch synthesized under deuterium‐labeled glucose/H_2_O condition showed similar signatures to those of deuterium‐labeled glucose themselves, indicating the dominating condensation mechanism from glucose to starch without breaking sugar rings (Figure S7G–I, Supporting Information).^[^ ^31^ ^]^ In contrast, synthesized starch from glucose isotopologues in 50% D_2_O/H_2_O solution showed mixed D‐sources from deuterium‐labeled glucoses and D_2_O (Figure S7J–L, Supporting Information).

Given that oilseed of *A. thaliana* contains abundant triglycerides, it is reasonable to speculate that newly synthesized starches are transformed from the existing oil stored in the dry seeds. In comparison to the direct condensation from external deuterated glucose to starch, the deuterium derived from D_2_O was incorporated into the C‐D bond via *de novo* synthesis, including β‐oxidation, the glyoxylate cycle, the tricarboxylic acid cycle (TCA cycle), and gluconeogenesis,^[^ ^24^, ^27^ ^]^ as illustrated in Figure 2G. This highlights the ability of C‐D imaging of SRS microscopy to probe metabolic flow, showcasing how storage oil transitions to starch during seed germination.

### 2.2 Imaging Metabolic Heterogeneity in the Radial Plane of Adult Plants

Next, the growth and development of the adult whole plants were studied, in which D_2_O serves as a better labeling precursor than deuterated macromolecules,^[^ ^30^, ^32^ ^]^ due to the following reasons. First of all, D_2_O directly participates in photosynthesis – the major source of organic matters.^[^ ^33^ ^]^ Second, D_2_O labels a diverse range of *de novo* synthesized metabolites such as lipids, proteins, and starches. Third, D_2_O diffuses and transports freely into cells without tissue bias,^[^ ^34^ ^]^ making it a universal tracer for most biochemical reactions. In the study of adult plants, *A. thaliana* plants were initially cultivated with ordinary water (H_2_O) from seed germination till the stems started to grow (needed ≈3 weeks), then water was switched to 50% D_2_O/H_2_O for isotope labeling. After culturing the adult plants with 50% D_2_O/H_2_O for ≈15 days, plant tissues were collected and treated with ethanol to remove chlorophyll (see Methods for details) to avoid interference of transient absorption signals, then transverse sections were obtained for SRS imaging.

**Figure 3:**
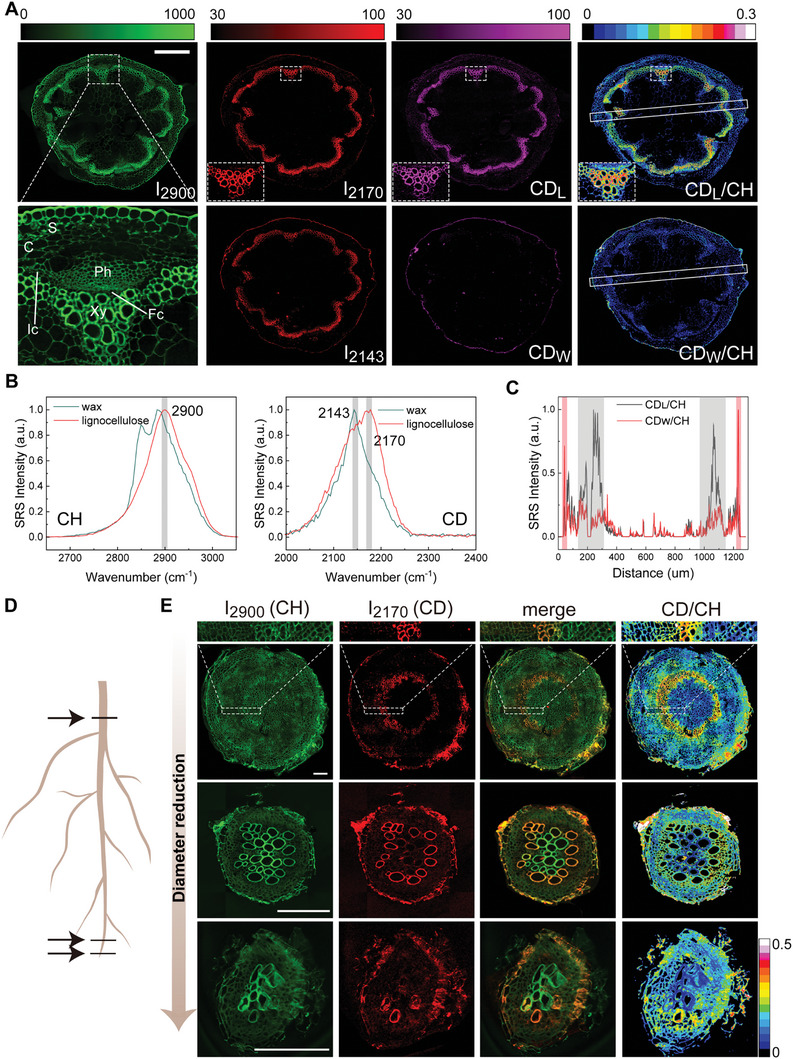
SRS microscopy reveals metabolic heterogeneity in the radial panels of adult *A. thaliana* plants. Plant watering was switched from H_2_O to 50% D_2_O/H_2_O at ≈3 weeks from seed germination, and tissues were collected after 15 days of D_2_O culturing. A) SRS images of stem cross‐sections at Raman frequencies of C‐H stretch (2900 cm^−1^, green); C‐D stretch (2170 and 2143 cm^−1^) before spectral decomposition (red), after spectral decomposition (magenta) into lignocellulose (CD_L_) and wax (CD_W_), and ratiometric images of CD_L_/CH (CD_W_/CH). The dotted rectangles are magnified regions. S, starch sheath; C, cortex; Ph, phloem; Xy, xylem; Fc, fascicular cambium; Ic, interfascicular cambium. B) Normalized SRS spectra in C‐H (C‐D) channel of wax and lignocellulose of cross‐sections of adult plants of *A. thaliana*. C) Intensity profiles of CD_L_/CH ratio (gray) and CD_W_/CH ratio (red) along the stripes in the fourth column in (A). The CD_L_/CH ratio (gray) shows a strong signal at secondary xylem mainly composed of lignocellulose; while the CD_W_/CH ratio (red) shows a strong signal in the outer layer of the epidermis rich in wax. D) Illustration of hypocotyl/root cross‐sections selected for imaging. E) SRS images of the cross‐sections taken with varying diameters of hypocotyl/root with C‐H band (2900 cm^−1^, green), C‐D band (2143 cm^−1^, red), overlay of C‐H and C‐D band and CD/CH ratio. Scale bars: 200 µm in (A), 100 µm in (D).

Imaging the transverse sections of the stem in the C‐H stretch region (2900 cm^−1^) captured detailed tissue structures (**Figure** **3**A, green), including cortex (C), phloem (Ph), xylem (Xy), fascicular cambium (Fc), interfascicular cambium (Ic), and starch sheath (S), aligning with previous findings.^[^ ^35^ ^]^ Interestingly, when imaged in the C‐D stretch frequency at ≈2143 and 2170 cm^−1^, only a few layers of xylem (mainly secondary xylem) and epidermis showed newly synthesized C‐D contents (Figure 3A, red). Using spectral analysis, it could be seen that xylem mainly exhibited signals of lignocellulose – major composition of cell walls,^[^ ^36^ ^]^ while epidermis showed strong signals of wax on its surface (Figure S8, Supporting Information).^[^ ^37^ ^]^ To verify chemical compositions, confocal Raman spectroscopy in the whole spectral range was performed (Figure S8, Supporting Information), showing the main peaks of lignin and cellulose (≈1100 cm^−1^, ≈1600 cm^−1^, and ≈2900 cm^−1^) in the cell walls of xylem. The characteristic peak of pectin (≈850 cm^−1^) was not obvious in samples of this work.^[^ ^11b^ ^]^ Since the molecular structure and Raman spectra of hemicellulose (xylan) are similar to that of cellulose, they were not separated in this study. On the other hand, Raman spectra of epidermis also verified abundance of wax. Moreover, substantial autofluorescence remained in plant tissues even after chlorophyll removal, which could interfere with spontaneous Raman measurements but not for SRS.

The C‐D signal of lignocellulose contributed to a Raman peak ≈2170 cm^−1^, corresponding to the CD_1_ vibration mode of the sugar ring; whereas the C‐D signal of the wax layer exhibited a peak ≈2143 cm^−1^, attributed to the aliphatic CD_1_ stretching (Figure 3B). These two distinct Raman bands allowed the identification of the two macromolecular components via spectral decomposition.^[^ ^24^ ^]^ CD_L_ and CD_W_ are termed to represent lignocellulose and wax, respectively. The CD_L_ image showed a distinct “annual ring”‐like pattern^[^ ^38^ ^]^ of the newly‐grown xylem (Figure 3A, magenta). This agrees with the fact that cambial cells frequently undergo proliferation and radial growth, resulting in cell wall thickening through differentiation into phloem and xylem cells, ultimately leading to secondary growth.^[^ ^35^, ^38^, ^39^ ^]^ The strong C‐D signal in secondary xylem is a direct evidence of its high metabolic activity. In contrast, cortical cells outside the cambium, pith cells inside the cambium, primary phloem, and primary xylem cells appear metabolically inactive without detectable signs of deuterium.^[^ ^39b^ ^]^ In the CD_W_ image (Figure 3A, magenta), stems exhibited newly‐synthesized wax in the outer layer of the epidermis. CD_L_/CH (CD_W_/CH) ratios were utilized to evaluate the extent of bio‐synthesis, as an indication of relative metabolic rate, and to quantify the metabolic activity of lignocellulose (wax) normalized against heterogeneity within the same section, showing significantly elevated metabolic activity in the secondary xylem and epidermis compared to other areas (Figure 3C).

Aside from the stems, cross‐sections of the underground hypocotyl/roots were also imaged (Figure 3D,E). Since wax in the epidermis is less prominent in roots, CD_L_ signal at 2170 cm^−1^ of xylem was the main target. A similar “annual ring”‐like pattern in hypocotyl and roots was observed in regions of secondary growth, including secondary xylem cells as deuterated structures, revealing their active metabolism before apoptosis. As the chosen cross‐sections gradually approaching the root tip with decreased diameters, the multi‐layered deuterated xylem transitioned to single‐layered and eventually to single‐celled appearances, signifying the very initiation of secondary growth and formation of developmental pattern in plant thickening. Within the same tissue, complementary signal strengths in the C‐H and C‐D channels of the vascular bundles were seen (Figure 3E), showing remarkable metabolic heterogeneity in plants at single‐cell level. Additional imaging results of the cross‐sections of stems and roots are shown in Figure S9 (Supporting Information).

### 2.3 Quantification of Metabolic Activities in the Axial Growth

**Figure 4:**
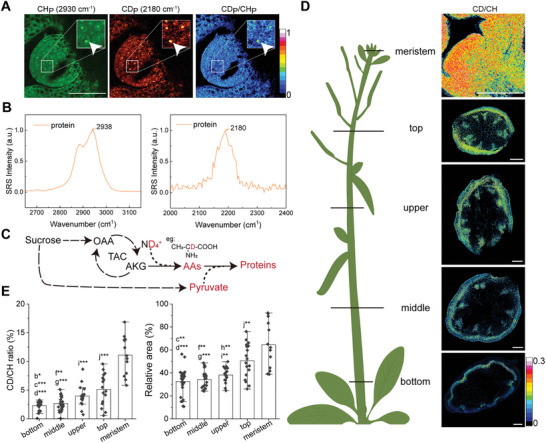
Detection of high metabolic activity of meristem in the stem tip and quantification of axial growth. A) SRS images of meristem of *A. thaliana* collected at CH_P_ channel (2930 cm^−1^, green), CD_P_ channel (2180 cm^−1^), and CD_P_/CH_P_ ratio without spectral decomposition. The magnified images showed abundant newly synthesized protein and individual cell nucleus. White arrowheads indicate the most condensed protein synthesis occurs in the nucleus. B) SRS spectra of protein in the dense nucleolus in (A) in the C‐H and C‐D regions. C) Metabolic diagram of protein synthetic pathways with deuterium transfer. D) SRS CD/CH ratiometric images of transverse cross‐sections of stems along the axial direction of the plant, from the tip to the bottom. E) Quantification of the mean CD/CH ratios (left) and relative areas (right) (means ± SDs) of the transverse cross‐sections at different heights. The mean ratios were obtained by measuring the mean CD/CH values in areas with a threshold of CD/CH > 0. The relative areas were calculated by dividing the area with CD/CH > 0.02 by the total area of CD/CH > 0. n = 21, 21, 18, 16 and 13 for the repetitions of the bottom, middle, upper, top and meristem, respectively. Statistically significant differences were represented using one‐way analysis of variance (ANOVA) test. Pairwise comparisons were as follows: a = bottom_middle, b = bottom_upper, c = bottom_top, d = bottom_meristem, e = middle_upper, f = middle_top, g = middle_meristem, h = upper_top, i = upper_meristem, j = top_meristem. ***, *p* < 0.001; **, *p* < 0.01; *, *p* < 0.05 in ANOVA test. Scale bars: 50 µm in (A), 100 µm in (D).

One of the most active metabolic regions in plants is the meristem located in stem tips, which is developed into subsequent floral meristems.^[^ ^40^ ^]^ Shoots, leaves, and flowers all develop from a group of stem cells known as the shoot apical meristem (SAM), with rapid cell divisions in both peripheral zone and rib meristem.^[^ ^40a^ ^]^ Using the same adult plants as in the last section, SRS images of vibratome‐sectioned SAM tissues readily show well‐arranged cells with morphologically distinct cytoplasm and nucleus in the C‐H channel (**Figure** **4**A). The C‐D specific images demonstrated newly synthesized biomass filled in the whole SAM region, indicating all the cells are undergoing active metabolism and proliferations. SRS spectra indicated the main biomolecular component in the SAM cells is intracellular protein, with featured Raman peaks matched with previous reports in the C‐H and C‐D spectral regions of ordinary protein (CH_P_) and deuterated protein (CD_P_)^[^ ^24^, ^30^ ^]^ (Figure 4B). The distributions of CD_P_/CH_P_ ratio in SAM cells reveal that the most condensed protein synthesis occurs in the nucleus (Figure 4A). The possible protein synthesis pathway was conjectured in Figure 4C.^[^ ^41^ ^]^

The continued division of cells in the rib meristem and the peripheral zone enables the SAM to grow upward and leave the aged cells behind, leading to the stem's growth in height, known as primary growth.^[^ ^40a^ ^]^ SRS imaging of tissue sections at different heights of the plant allowed directly visualize the change of metabolic activities along the axial direction using the CD/CH ratio (Figure 4D). Metabolic activity appears gradually enhanced from the bottom of the stem toward the tip, with an increasing trend of the occupied area ratio of the newly synthesized deuterated biomaterials (Figure 4D). This could be quantified as the mean values of the CD/CH ratio at different heights (Figure 4E, left). Additionally, we calculated a ratio of the area with CD/CH > 0.02 divided by the total area of CD/CH > 0, as an indicator of high metabolic activity (Figure 4E, right) (see Experimental Section for details). These results confirmed that increased metabolic activity takes place in the stem with higher heights and reaches the maximum at the meristem tip, consistent with the principle of primary growth. As a control, quantification of pure H_2_O cultured plants of *A. thaliana* showed negligible C‐D signal (Figure S10, Supporting Information).

### 2.4 Imaging oil Formation and Storage in the Seed Development Stages

**Figure 5:**
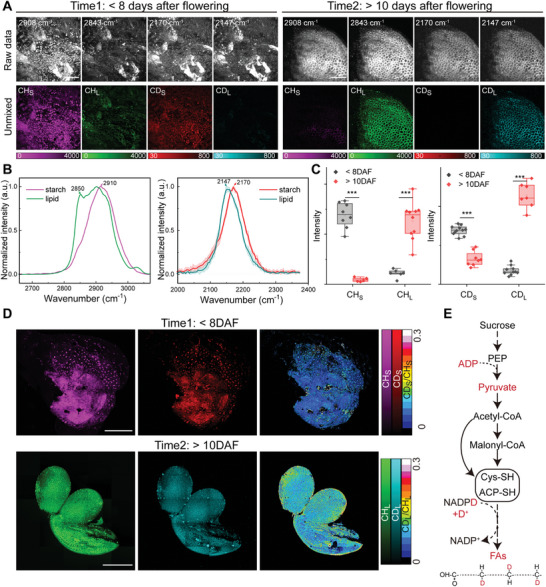
Metabolic pathways of oil storage in the seed development process. A) SRS images of new seeds at different stages (< 8 DAF and > 10 DAF), showing raw images before spectral unmixing (upper) and decomposed distributions (bottom) of lipid (CH_L_, CD_L_) and starch (CH_S_, CD_S_). B) Normalized C‐H (left) and C‐D (right) SRS spectra of lipid and starch measured in the new seeds. C) Quantification of the mean SRS signal intensities (means ± SDs) of lipid and starch in the C‐H and C‐D channels of developing seeds at the two stages. The mean SRS intensities are obtained from decomposed concentration maps with MATLAB by measuring the mean gray intensities of the area with SRS intensity > 0. *** indicates *p* < 0.001 in *t*‐test. For the stage < 8 DAF, n = 8, 6, 11, 10 for CH_S_, CH_L_, CD_S_, CD_L_; For the stage > 10 DAF, n = 5, 10, 6, 7 for CH_S_, CH_L_, CD_S_, CD_L_, respectively. D) SRS images of seeds at the two stages. The CD/CH ratio images were used to compare the metabolic intensities between the two different stages. E) Metabolic diagram of lipid synthesis pathways with possible deuterium transfer. Scale bars: 50 µm in (A), 200 µm in (D).

In addition to the earlier studies in the seed germination process, the metabolic activities during seed development process were also imaged. Under the same 50% D_2_O/H_2_O watering strategy as in the previous section, seeds of *A. thaliana* were harvested at two different stages based on the differentiation degree of columellae in the mucilage cells: <8 days after flowering (<8 DAF) and >10 days after flowering (>10 DAF).^[^ ^42^ ^]^ Spectral images were decomposed into separated starch and lipid channels in the C‐H and C‐D regions (**Figure** **5**A), revealing the content and distribution of macromolecules in different stages of seed development. In the early stage of <8 DAF, enriched starch granules were formed with a distinct C‐D spectrum peaked at 2170 cm^−1^. However, in the >10 DAF stage, lipids took over starch as the main biocomponent (Figure 5A), exhibiting a separate C‐D Raman peak at 2147 cm^−1^ (Figure 5B). The C‐D spectra align well with a previous literature,^[^ ^30^ ^]^ assigned as the CD_1_ vibrational mode due to sparse labeling of deuterium.^[^ ^24^ ^]^ The statistical analysis of the mean intensities of starch and lipid (Figure 5C) indicates a metabolic pathway from starch to storage oil during the seed development process, which is opposite to that observed during seed germination process (Figure 2).^[^ ^42^ ^]^ These finding are consistent with previous studies using iodine staining^[^ ^42^ ^]^ or gas chromatography‐mass spectrometry.^[^ ^27a^ ^]^ SRS provides much more direct optical visualization of the metabolite with high spatial resolution and minimal tissue invasion (Figure 5D). Consequently, SRS microscopy has enabled tracing deuterium along lipid synthesis from deuterated glucose or water, with pathways shown in Figure 5E.^[^ ^43^ ^]^

### 2.5 Deuterium Transfer from *A. Thaliana* to Aphids

**Figure 6:**
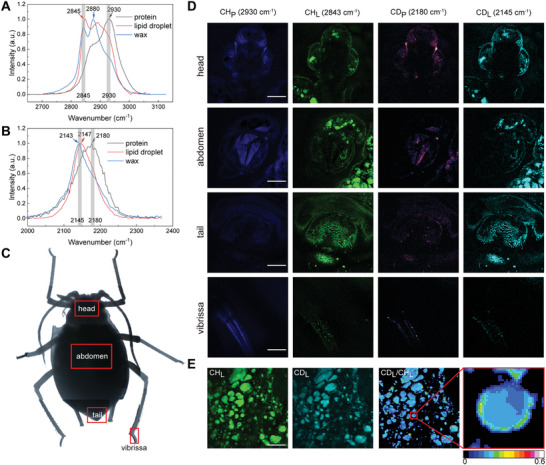
Metabolic flow from *A. thaliana* to aphids. A) Normalized SRS spectra of protein (gray), lipid droplet (red), and wax (blue) in the C‐H stretch region. B) Normalized SRS spectra in the C‐D stretch region. All spectra were extracted from different organs of aphids. C) Bright light image of an aphid. D) Unmixed SRS images of different organs corresponding to the red boxes in (C). E) SRS images of lipid droplets collected from CD_L_ and CH_L_ channels, and image of CD_L_/CH_L_ ratio. The magnified image shows heterogeneous distribution of deuterium intensity within a single droplet. Scale bars, 50 µm.

Aphids, specialized herbivores that feed on the phloem of vascular plants, can ingest deuterated secondary metabolites and water present in the phloem sap during feeding. These deuterated compounds may then be transported across the gut membrane into the aphids' haemocoel and participate in subsequent metabolic processes.^[^ ^44^ ^]^ By studying aphids feeding on deuterated *A. thaliana*, the metabolic flow of deuterium atoms from plants to animals could be visualized. The author observed three types of C‐D spectra in different regions of aphid tissue: lipid droplet, wax, and protein (**Figure** **6**A,B), which showed the same C‐D Raman spectra as those found in plants (Figures 3, 4, and 5B). Among them, the lipid droplet and wax have very similar spectra (peaked ≈2145 cm^−1^) and are classified as lipids, while the spectral peak of protein is located ≈2180 cm^−1^. SRS images of a variety of organs were obtained and decomposed into lipid and protein distributions (Figure 6D), such as vibrissa, head, tail and abdomen, showing distinguishable metabolic features. The head and tail exhibit efficient lipid synthesis, with enriched lipid droplets and waxy villi. Whereas the abdomen shows active protein synthesis. This showcases a metabolic flow model from plants to animals that can be generally visualized by SRS microscopy. Moreover, intensity ratio between C‐D and C‐H channels (CD_L_/CH_L_) reveals heterogeneous distributions of deuterium enrichment within individual lipid droplets of fat bodies in abdomen synthesized in aphids (Figure 6E), agreeing with previous observations in Drosophila.^[^ ^45^ ^]^

## 3 Discussion and Conclusion

SRS microscopy integrated with deuterated water offers an efficient optical means to learn the distributions and fates of deuterium involved in plant metabolism. Unlike previous works focusing on specific metabolic pathways,^[^ ^3b–d^ ^]^ our study provided comprehensive pictures of plant biological activities that take hydrogen atoms from water to build or rebuild organic compounds, such as sugar, lipid, protein, etc. Comparing with D_2_O‐associated biochemical processes in animals, plant photosynthesis transfers deuterium/hydrogen to glucose with 100% efficiency, which further serves as substrates for downstream biochemical reactions along with D_2_O itself. Note that relatively high concentrations of D_2_O were utilized in the current proof‐of‐concept work, which could be reduced with improved detection sensitivity for low‐concentration C‐D bonds using frequency modulation technique or deep‐learning denoising algorithms.^[^ ^46^ ^]^ Moreover, sucrose may be preferred over glucose as isotopologues for future studies because it is more physiological for plants.

For the metabolic switch between seed germination and seed development, although previous works have confirmed the interconversion between sugar and oil,^[^ ^47^ ^]^ direct visualization of the processes is reported for the first time, as far as the authors know. Although traditional techniques of metabolite detection and identification such as MS, NMR, or electrochemistry have laid a solid foundation for the establishment of functional metabolomics,^[^ ^3a^ ^]^ SRS offers chemical imaging with high spatial resolution in a nondestructive way to assist the studies of plant metabolism and biomass allocation. Future researches may be extended to image the metabolic flows of other elements such as carbon,^[^ ^48^ ^]^ which could be used to gauge assimilation capacities, growth rates, and net primary production through photosynthesis, and to estimate ecosystem carbon budgets. Also, potential studies of nitrogen may be performed to learn more about nitrogen cycling and interaction between plants and mycorrhizal fungi, climate, and microbial.^[^ ^49^ ^]^

It is worth mentioning that usually fresh unprocessed plant tissues could be imaged with SRS, and the chlorophyll clearing process is not required for most organs in *A. thaliana* except organs rich in chlorophyll such as the leaves. Autofluorescence of plant tissues does not affect SRS detection, but transient absorption (TA) signal of chlorophyll is simultaneous detected in SRS imaging. For spatially separated chlorophyll, cellulose, and wax, etc., it is convenient to differentiate these chemical components via hyper‐spectral SRS microscopy based on their distinct spectral features.^[^ ^50^ ^]^ However, in leaves where synthesized starch colocalizes with chlorophyll in chloroplast, TA signal of chlorophyll may overwhelm SRS signal of starch, hindering the quantitative identification of starch granules. After chlorophyll removal, SRS could successfully unveil starch granules and protein‐rich stroma inside chloroplasts (Figure S11, Supporting Information). Besides, advanced optical technique such as frequency‐modulated (FM) SRS^[^ ^51^ ^]^ may be applicable to extract starch from chlorophyll in leaves to further investigate carbon storage via photosynthesis.

Pulse‐chase experiments integrated with isotope‐traced SRS microscopy could provide further insights into the metabolic details. In the current study, H_2_O was switched to D_2_O/H_2_O mixture in a fixed time point during plant growth, which readily allowed us to differentiate the newly synthesized biomass in the metabolically active areas (such as the secondary xylem) from the existing inactive areas (cortical cells outside the cambium, pith cells inside the cambium, primary phloem, primary xylem cells, etc.) in the stem cross‐sections of adult plants. More sophisticated pulse‐chase strategies can be planned to focus on specific developmental processes. For instance, the D_2_O/H_2_O switching/tracing points may be set during the seed development stage, which could enable the visualization of starch‐oil conversion with more dynamic and spatial information. In a bigger picture, this technique may even quantify and compare the energy harvesting efficiencies for different plant organs at different growth stages.

Further technical improvements of SRS may provide unique platforms for plant studies, ranging from more detailed metabolism of specialized organs using spectral analysis to in vivo imaging of water transportation in vascular tissue and water absorption capacity of apical meristem. The non‐invasive technique allows imaging of living tissues. Challenges exist due to the limited optical imaging depth of SRS for thicker plant tissues and the strong absorption of chlorophyll in the transmission detection mode. Optimization of epi‐detected SRS for plant imaging and the envision of handheld probes may offer possibilities for data collection directly in the farm fields.

In summary, deuterium‐traced stimulated Raman scattering microscopy has been applied to image the metabolic activities and monitor hydrogen flows at different developmental stages of *A. thaliana*. By detecting newly synthesized biomolecules, SRS microscopy provides valuable insights into various metabolic processes of plants, including seed germination and development, secondary growth, primary growth, etc. Furthermore, the presence of C‐D signal in aphids feeding on *A. thaliana* highlights the metabolic flow of deuterium from plants to animals. Our method holds potentials to investigate the genesis, consumption, and degradation of biomass in plants, as well as study plant‐animal interactions, and explore other metabolic flows such as carbon fixation.

## 4 Experimental Section

### Materials

Metabolic labeling was achieved with D_2_O (99 atom% D, Aladdin, China). Spectra were assigned using standards including fatty acids, oleic acid‐d9 (Sigma‐Aldrich); protein, bovine serum albumin (BSA) powder (Fraction V, Aladdin, China); glucose, glucose‐d1‐1 (Aladdin, China), glucose‐d2‐6,6 (Sigma‐Aldrich), glucose‐d7‐1,2,3,4,5,6,6 (MACKLIN); and cellulose (Aladdin, China), starch (Aladdin, China), paraffin (Aladdin, China), xylan (Aladdin, China, from corn cob), coniferyl alcohol (Aladdin, China).

### Spontaneous Raman Spectroscopy

Spontaneous Raman scattering spectra were acquired on a home‐built Raman spectrometer, including a monochromator (iHR320, Horiba), a charge‐coupled device camera (Symphony, Horiba), and a microscope (IX71, Olympus) with a 40 × air objective with 633 nm helium‐neon laser beam at room temperature. 10 s acquisition time accumulated by 10 was used to collect Raman spectra of all samples at a single point under identical conditions. Quartz plate was used to suppress autofluorescence generated by normal glass slide.

### Stimulated Raman Scatter (SRS) Microscopy

SRS experiments were performed on the home‐built system. For the detail of SRS microscope, a commercial femtosecond laser system (Insight DS+, Spectra‐Physics) produced two synchronized pulse trains at 80 MHz. The fixed fundamental output of 1040 nm was employed as the Stokes beam (≈200 fs), while the tunable optical parametric oscillator output (680 to 1300 nm, ≈150 fs) served as the pump beam. To acquire high spectral resolution (≈13 cm^−1^), pulse durations of the pump and Stokes beams were chirped and stretched by passing through SF57 glass rods (≈3.8 ps for the pump pulse and ≈1.8 ps for the Stokes). The intensity of the stokes beam was modulated at 1/4 of the laser pulse repetition rate (80 MHz) using a polarizing beam splitter (PBS) and an electrooptical modulator (EOM, EO‐AM‐R‐20‐C2, Thorlabs). The two laser beams were spatially and temporally overlapped via a dichroic mirror, then delivered into a laser scanning microscope (FV1200, Olympus) equipped with galvo mirrors for raster scanning. The combined beams were focused onto the sample by a 60 × water immersion objective lens (Olympus, UPLSAPO 60XWIR, NA 1.2). The transmission of the forward‐going pump and Stokes beams was collected by a high N.A. oil condenser (oil immersion, NA = 1.4, Nikon) after passing through sample. Transmitted through a bandpass filter (CARS ET890/220, Chroma), the stimulated Raman loss (SRL) signal was detected by a homemade reverse‐biased photodiode (PD) and demodulated with a lock‐in amplifier (HF2LI, Zurich Instruments) at 20 MHz to feed the analog input of the microscope to form images. The Raman band of C‐H bonds was recorded at the pump laser of 802 nm, while the signal of C‐D bonds was measured with the tuned 850 nm pump beam. All the images used the same setting of 512 × 512 pixels with a pixel dwell time of 2 µs. The lateral resolution of the microscope system is ≈350 nm, and the depth resolution is ≈1 µm. To image a large area of tissue, mosaicking, and stitching were performed to merge the small fields of view into a large flattened image. Laser powers at the sample were kept as: pump 30 mW and Stokes 40 mW.

### Seeds Growth Conditions

The seeds used in this work are dry seeds of wide type *A. thaliana*. All seeds were surface‐sterilized, sown on liquid MS plates, and vernalized at 4 °C for 2 days before growing at a constant temperature of 22–23 °C in a growth incubator for 7–9 days. To make seed medium containing D_2_O, a mixture of D_2_O (Aladdin, China) and distilled H_2_O was used to dissolve MS powder (Sigma) and then sterilized the medium by filtering. The MS media were supplemented with exogenous sucrose. Seeds were germinated in dark. For quantitative analysis, the media were prepared with different concentrations of D_2_O (0%, 25%, 50%, 75%, 100% [v/v]). For the purpose of spectra tracing, the exogenous sucrose was replaced by glucose‐d1‐1 (Aladdin, China), glucose‐d2‐6,6 (Sigma‐Aldrich), glucose‐d7‐1,2,3,4,5,6,6 (MACKLIN), respectively.

### Plants Culture and Labeling

All experiments were carried out with *Arabidopsis thaliana*, ecotype Columbia (Col‐0) in a controlled environment room under a 16‐h‐light/8‐h‐dark regime. 7‐day‐old seedlings (cultured with H_2_O) were transplanted to soil, and then grown on individual pots of fertilized bedding compost for about three weeks with regular watering with H_2_O, then for two weeks with 50% [v/v] D_2_O for labeling. The relative humidity was 60% and the temperature was 23 °C.

### Plant Tissue Section Preparation for Imaging

The plant tissues were fixed in FAA fixative solution (3.7% formaldehyde, 5% HAc, 50% ethanol) for 24 hours at 4 °C. Then the fixed tissue was dehydrated in a graded series of ethanol (75%, 90%, 100%, each step 30 minutes) for chlorophyll clearing. Then these tissues were dissected in a drop of chloral‐hydrate solution (chloral‐hydrate: H_2_O: glycerol = 8:2:1) for transparency. After treatment and labeling, stems and roots were radically sliced into thin slices by hands. Meristem in the stem tip was sliced using a vibratome. The tissue slices were collected and sealed between glass slide and coverslip.

### Two‐Component Chemical Unmixing

Details for the two‐component unmixing was described previously.^[^ ^24^ ^]^

### Imaging Processing and Statistical Analysis

All steps of image process used ImageJ. For SRS images of CD and CH channels, corresponding off resonance images were subtracted from the on‐resonance images. For obtaining ratiometric images, MATLAB was used to generate the mask images. Statistical analysis and visualization were done in Origin and Excel.

### Indicator of High Metabolic Activity

CD/CH ratios were used as an indicator of metabolic activity. The CD/CH ratio can be calculated using Equation 1.^[^ ^24^ ^]^

(1) $$\;\frac{CD}{CH}=\frac{a*\left[C-D\right]}{b*\left[C-H\right]}$$

*a* and *b* are factors that transform chemical bonds concentrations at different wavenumber regions into SRS microscopic signals intensity; [*C* − *D*] and [*C* − *H*] are the concentrations of D‐labeled and H‐labeled chemical bonds, respectively. To calculate the coefficient *a*/*b*, glucose and its isotopologue glucose‐d7‐1,2,3,4,5,6,6 was used as two standard samples. Their SRS spectra are displayed in Figure S12 (Supporting Information) and the ratio of integrated spectrum was obtained as $\frac{CD}{CH}\approx 0.348$, showing different conversion efficiency between high wavenumber region and cell‐silent spectral region. As [*C* − *D*] equals to [*C* − *H*] in the two isotopologues, the coefficient *a*/*b* can be calculated as 0.348.

In deuterium ‐labeled plants, Equation (1) can be rewritten as Equation (2).^[^ ^24^ ^]^

(2) $$\;\frac{CD}{CH}=\frac{a*\left[C-D\right]}{b*\left[C-H\right]}\;=\frac{a*\left[new\right]*G}{b*\left(\left[new\right]*\left(1-G\right)+\left[old\right]\right)}\;=\frac{a}{b}\;*\frac{G}{\frac{\left[total\right]}{\left[new\right]}-G}$$

[old] is the number of biomolecules that present before deuterium‐labeling and remained at imaging time; [new] is the amount of newly synthesized biomolecules till the time of imaging; [total] = [old]+[new]; and *G* is the ratio of deuterium‐labeled biomolecules to the newly synthesized biomolecules. In this experiment a 50% D_2_O/H_2_O medium was used so $G\leq 0.5\ll \frac{\left[total\right]}{\left[new\right]}$. Then Equation 2 can be simplified as the following.

(3) $$\;\frac{CD}{CH}=\frac{a}{b}*G*\frac{\left[new\right]}{\left[total\right]}$$

The study defines $\frac{\left[new\right]}{\left[total\right]}>10\%$ as an indicator of high metabolic activity. Therefore, a high metabolic activity can be indicated when $\frac{CD}{CH}\approx 0.348*0.5*\frac{\left[new\right]}{\left[total\right]}>0.0174∼0.02$.

### Statistical Analysis

Independent experiments or repeated measurements were conducted for all data. Data for statistical analysis are shown as mean ± standard deviation (SD). Statistical analyses were performed using Excel (Microsoft 365, USA). Comparisons between two groups were performed using a two‐tailed student's *t*‐test. For multiple‐group comparisons, one‐way analysis of variance (ANOVA) was used to determine whether there were differences among the groups. A P value less than 0.05 was considered statistically significant.

## Conflict of Interest

The authors declare no conflict of interest.

## Author Contributions

S.B., J.A., and T.J. contributed equally to this work. M.J. designed the study. T.J., Y.Z., and X.Z prepared the samples. S.B. and J.A. performed stimulated Raman scattering microscopy experiments. S.B., J.A., and M.J. wrote the manuscript with contributions from all the authors.

## Supporting information



Supporting Information

Supporting Information

## Data Availability

The data that support the findings of this study are available from the corresponding author upon reasonable request.
