# Supplementary material for: Imaging Metabolic Flow of Water in Plants with Isotope‐Traced Stimulated Raman Scattering Microscopy
Source: Adv Sci (Weinh). 2024 Sep 20;11(42):2407543. doi: 10.1002/advs.202407543 (PMC11558102; doi:10.1002/advs.202407543)
Supplement: Supplementary file 1 — Supporting Information [file ADVS-11-2407543-s001.pdf]

ADVANCED SCIENCE
Open Access

Supporting Information

for *Adv. Sci.*, DOI 10.1002/advs.202407543

Imaging Metabolic Flow of Water in Plants with Isotope-Traced Stimulated Raman Scattering Microscopy

*Simin Bi, Jianpeng Ao, Ting Jiang, Xianmiao Zhu, Yimin Zhu, Weibing Yang, Binglian Zheng and Minbiao Ji**

Imaging Metabolic Flow of Water in Plants with Isotope-Traced Stimulated Raman Scattering Microscopy

Simin Bi[1#], Jianpeng Ao[1#], Ting Jiang[2#], Xianmiao Zhu[3,4], Yimin Zhu[3,4] , Weibing Yang[3,4], Binglian Zheng[2], Minbiao Ji[1*]

[1]State Key Laboratory of Surface Physics and Department of Physics, Academy for Engineering and Technology, Human Phenome Institute, Key Laboratory of Micro and Nano Photonic Structures (Ministry of Education), Yiwu Research Institute of Fudan University, Fudan University, Shanghai 200433, China

[2]State Key Laboratory of Genetic Engineering, Institute of Plant Biology, School of Life Sciences, Fudan University, Shanghai 200438, China

[3]National Key Laboratory of Plant Molecular Genetics, CAS Center for Excellence in Molecular Plant Sciences, Institute of Plant Physiology and Ecology, Chinese Academy of Sciences, Shanghai 200032, China

[4]CAS-JIC Center of Excellence for Plant and Microbial Sciences (CEPAMS), Institute of Plant Physiology and Ecology, Chinese Academy of Sciences, Shanghai, China.

***CORRESPONDENCE:** minbiaoj@fudan.edu.cn

Supplementary Materials

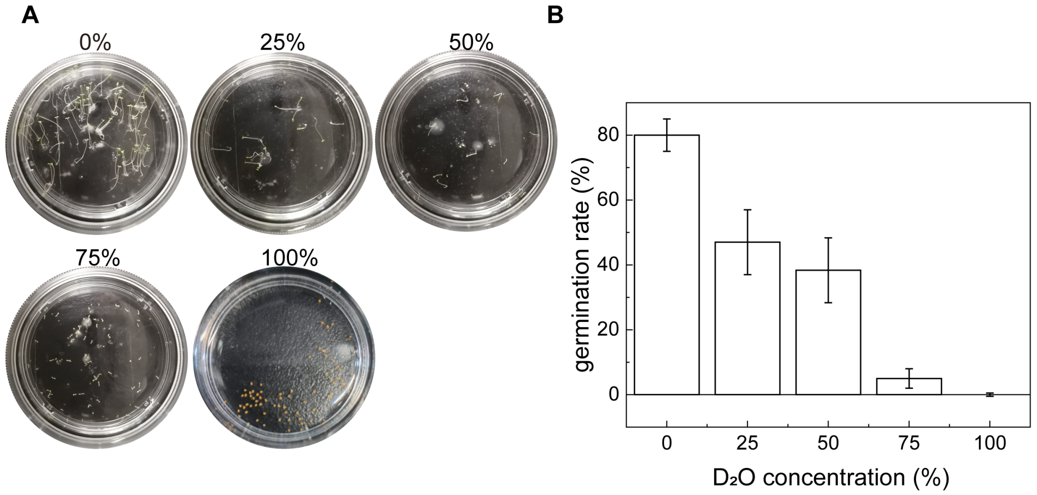

Figure S1. Toxicity of D_2O on seed germination. (A) The germination rate of seeds at the same time with different concentration on D_2O. **(B)** Statistics of mean germination rate (means ± SDs) on concentration of D_2O. The germination rates of seeds are about 80% with 0% D_2O concentration, 40% with 50% D_2O concentration and drops sharply with more than 50% D_2O concentration in our work. Since a high enough germination rate is needed, the D_2O concentration should be controlled no more than 50%. Combined with the linear relationship between SRS signal intensities at C-D channel and D_2O concentration shown in Fig. S2, the C-D signal is barely visible with D_2O concentrations under 25%. Therefore, 50% D_2O concentration was chosen not only for an acceptable germination rate but also for a clear enough signal. 50% D_2O does have a certain effect on the germination rate of seeds, but it can be seen from Fig.S2 that it does not have an essential effect on cell morphology and metabolites. Therefore, we believe this concentration (50%) will not change the essential metabolic activity during seed germination. In the next work, however, the D_2O concentration should be reduced slightly since the CD signal is obvious enough.

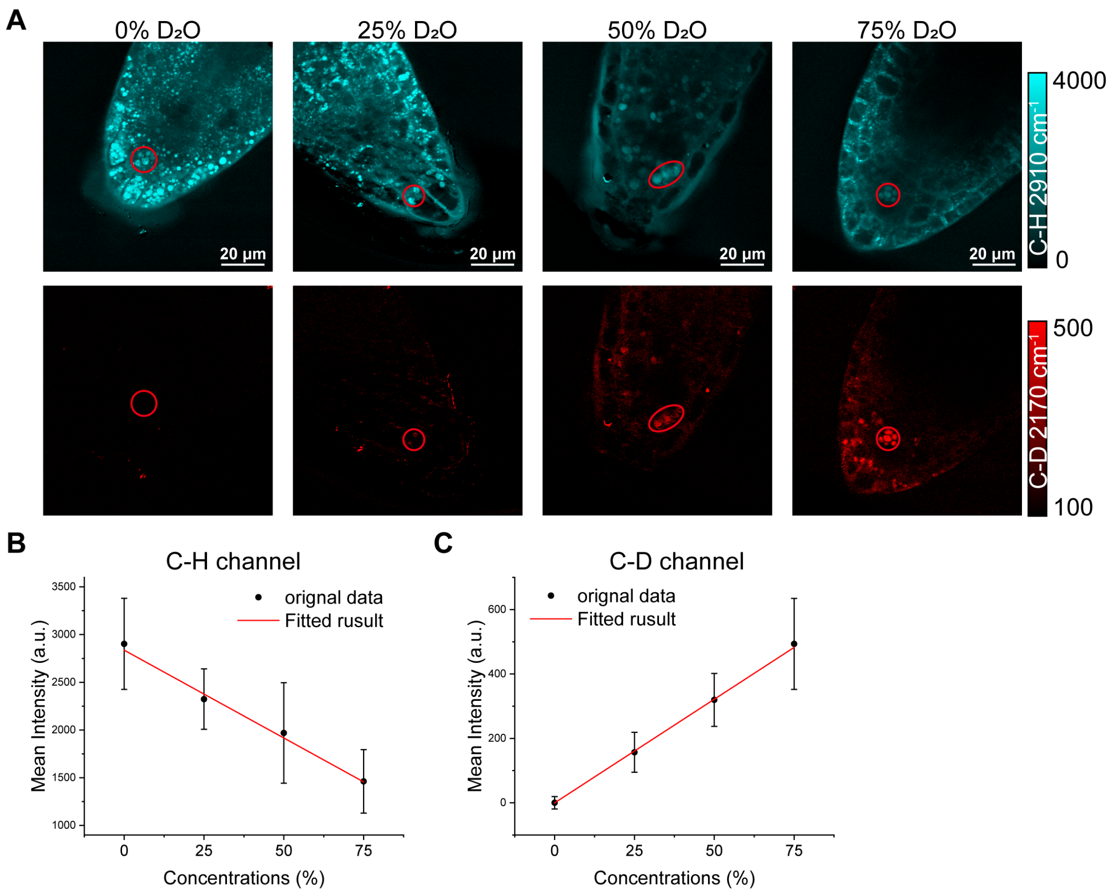

Figure S2 Quantification of newly-synthesized starch at various concentrations of D_2O. **(A)** SRS images of root tip of radicles cultured with different concentrations of D_2O at C-H frequencies (upper) and C-D frequencies (bottom). Red circles in each frame denote the position of starch. From left to right the D_2O concentrations are 0%, 25%, 50% and 75% in order. **(B)(C)** Linear fittings of dependence of mean SRS intensities (means ± SDs) of starch on concentration of D_2O at C-D and C-H channel. n = 15 for 0% D_2O; n = 35 for 25% D_2O; n = 65 for 50% D_2O; n = 45 for 75% D_2O.

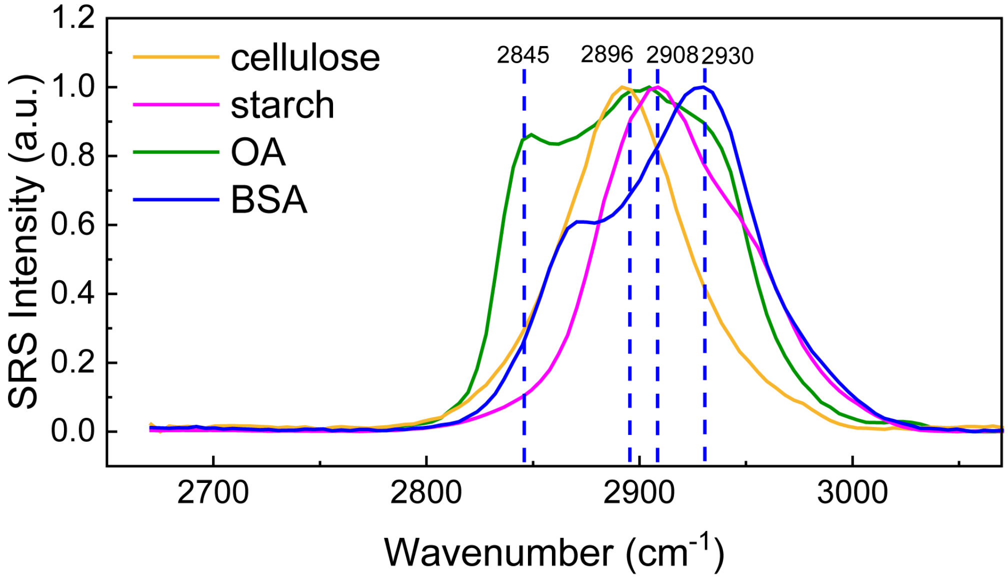

Figure S3. Normalized Raman spectra in C-H vibration region of four standard samples: cellulose, starch, oleic acid and bovine serum albumin.

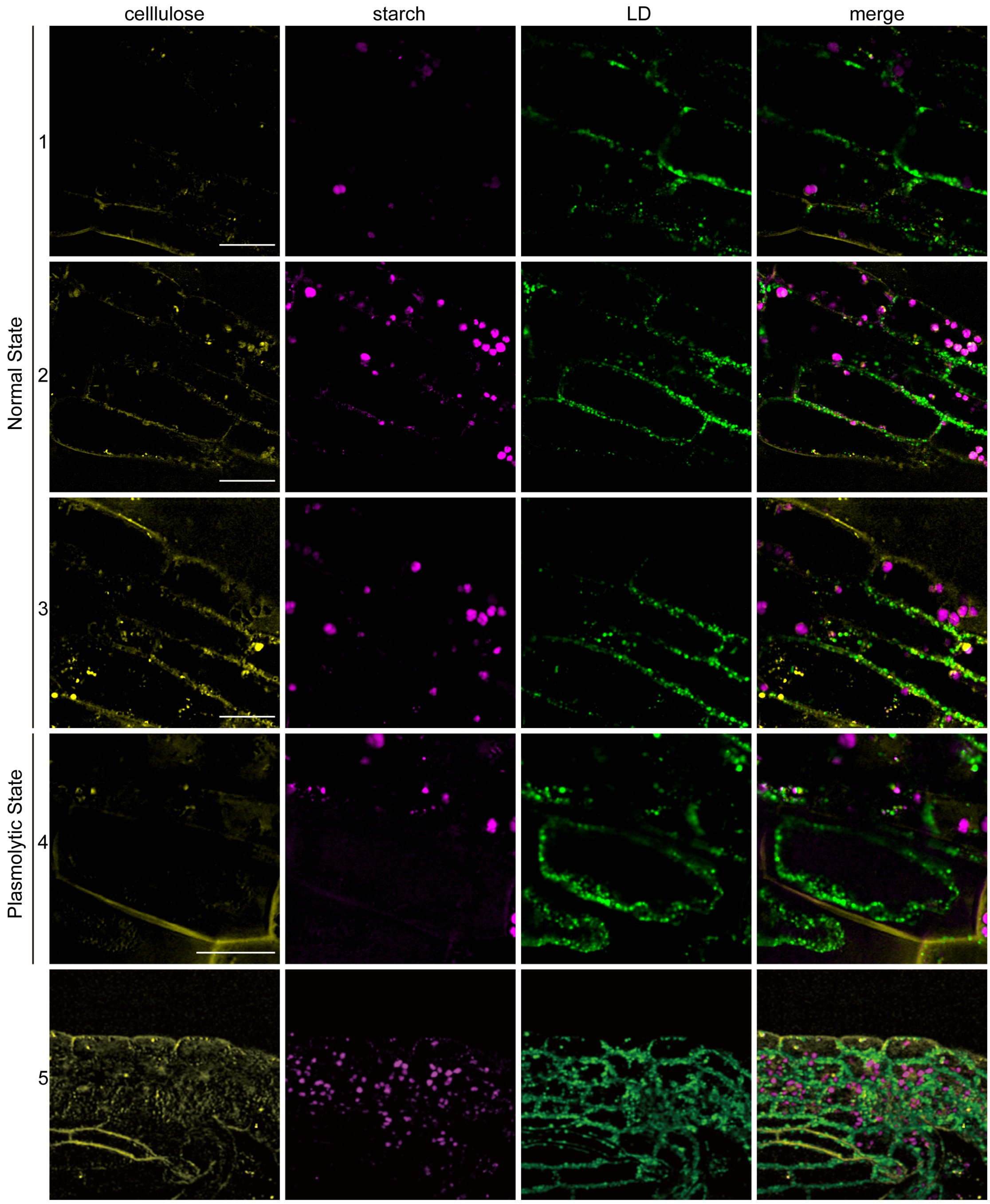

Figure S4. MCR reconstructed images of the radicles of four seedlings. MCR reconstructed images from columns one to four are: cellulose (yellow) which denotes cell walls since cellulose is a major component of cell walls, starch (magenta) which denotes starch granules inside plant cells, LD (green) which denotes lipid droplets inside plant cells and attached to the cell membranes, and their overlay merged image. The first three rows were collected from three samples of normal seedlings respectively. The seedling which is plasmolytic in the fourth row demonstrates that the lipid droplets are attached to the cell membrane. When the cell membrane adheres tightly to the cell wall, lipid droplets attached to the cell membrane overlap with the

cell wall in space (seedlings in the first three rows), which to some extent makes it difficult to unmix. The present work focuses more on lipid and starch of seedlings with less attention on the cellulose in the cell wall. Scale bars, 20 μm.

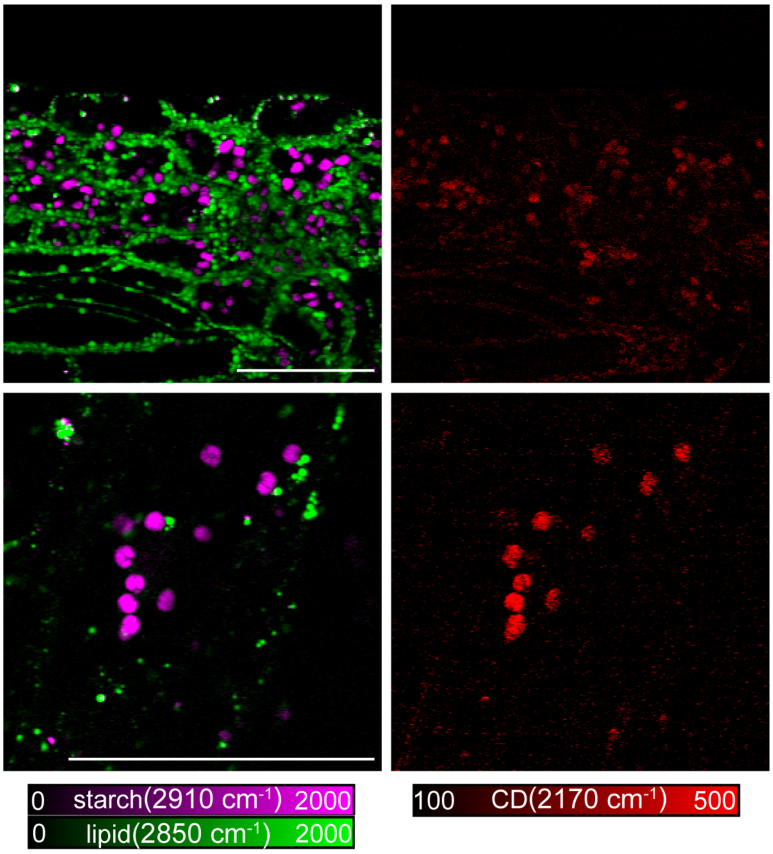

Figure S5. Two samples of seedlings cultivated with 50% D_2O/H_2O show C-D signal only in starch granules (red), but not in lipid droplets (green). Left, overlay image of lipid (2850 cm^{-1}, green) and starch (2910 cm^{-1}, magenta) in C-H channel obtained by hyperspectral SRS imaging and MCR decomposition. Right, SRS images in C-D channel (2170 cm^{-1}) showing D_2O-synthesized starch. Scale bars, 50 μm.

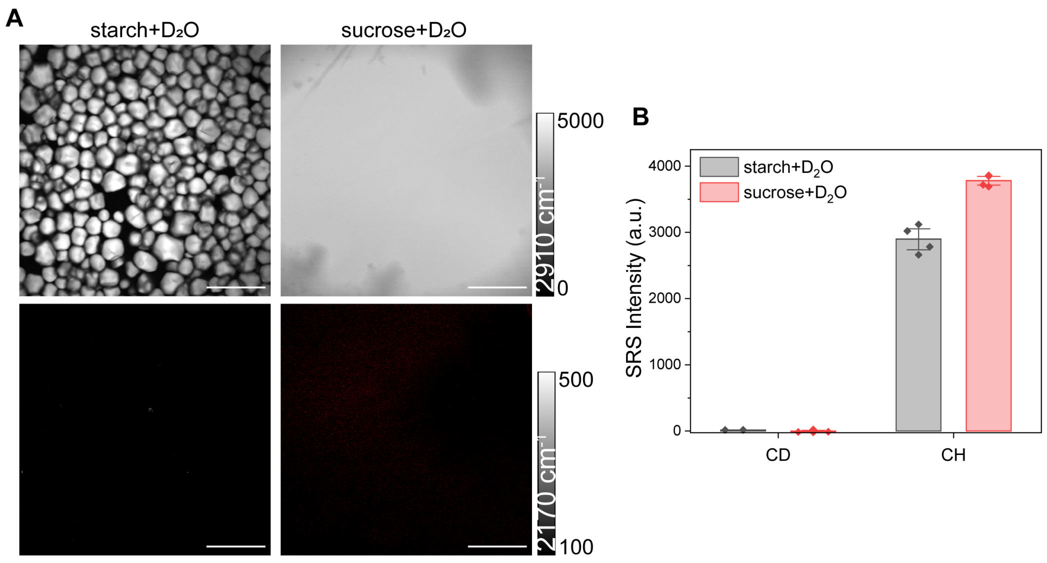

Figure S6. SRS intensity of starch and sucrose dissolved in D_2O. Starches and sucrose were dissolved in D_2O and rested for around 7 days to confirm that the C-D bonds were indeed originated from enzyme-catalyzed chemical reactions rather than non-enzymatic H/D exchanges. **(A)** Left, SRS images of mixtures of D_2O and starch at C-H channel (upper, 2910 cm^{-1}) and C-D channel (down, 2170 cm^{-1}); right, SRS images of D_2O and sucrose at C-H channel (upper, 2910 cm^{-1}) and C-D channel (down, 2170 cm^{-1}). No detectable C-D signal of both starch + D_2O and sucrose + D_2O could be observed, indicating no C-D bond formation through non-enzymatic H/D exchanges. **(B)** Quantification of SRS signal intensity in C-H and C-D channel showing no obvious C-D signal both in starch + D_2O and in sucrose + D_2O (n = 4 for each condition). Scale bars, 50 μm.

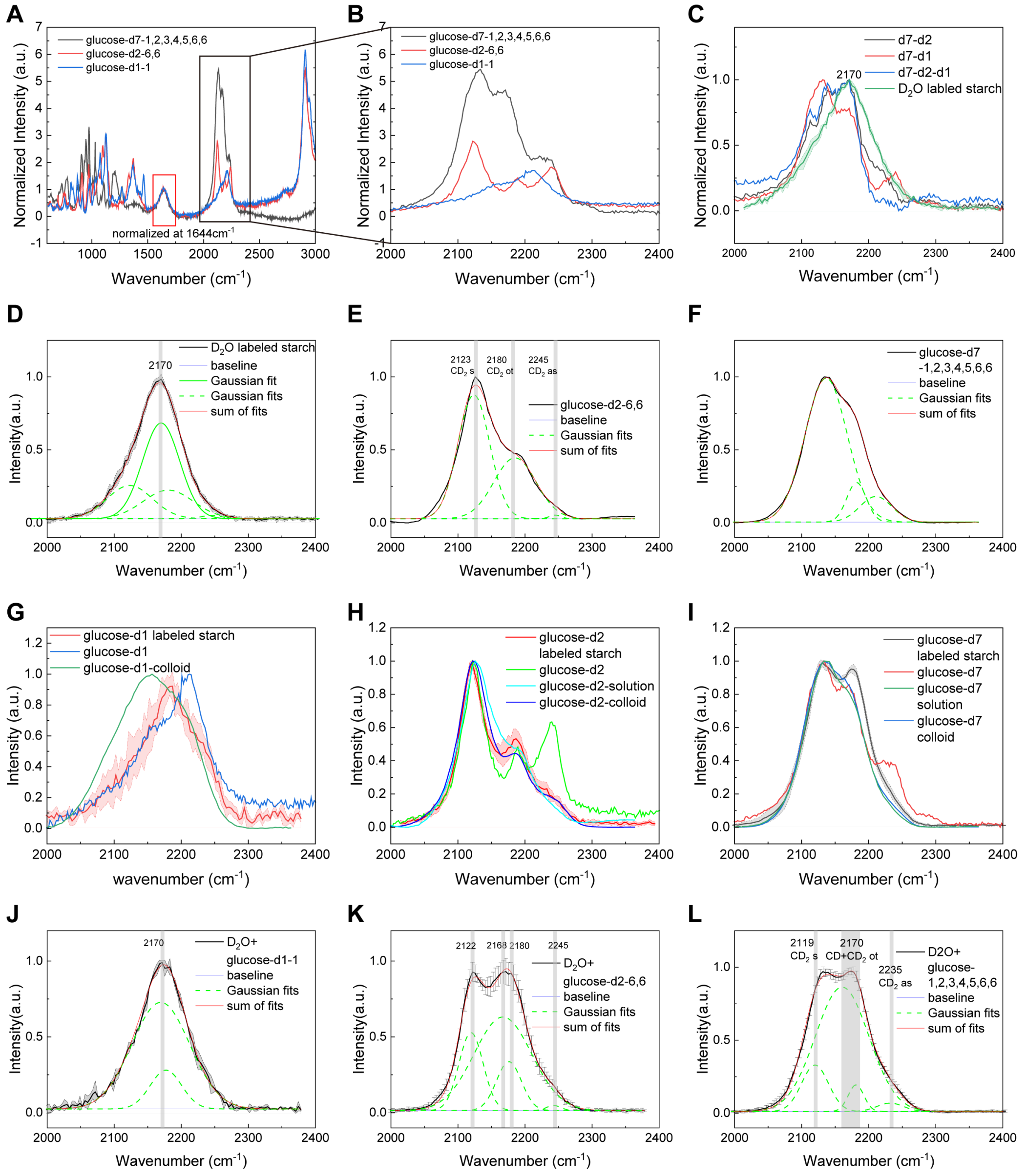

Figure S7. Raman spectra obtained for carbohydrate and spectral assignment. (A) Spontaneous Raman spectra for three glucose isotopologues normalized at 1644 cm^{-1} which assigned to HOH bending. [5] **(B)** Magnified C-D Raman spectra in (**A**). **(C)** Normalized Raman spectra obtained from subtraction of spectra of glucose-d1 (red), glucose-d2 (black), and both (blue) from that of glucose-d7. Raman spectra of three glucose isotopologues are shown in (**A, B**). SRS spectra of D_2O labeled starch is displayed for comparison (green), showing the fate of D1-D5 of D_2O-derived deuterium. **(D)** Normalized SRS spectrum of D_2O labeled starch and fitted by multiple Gaussian showing main contribution from CD mode (2170 cm^{-1}, green solid line) and minor contribution from CD_2 modes (green dotted lines). **(E)(F)** Normalized SRS spectra of glucose isotopologues and fitted by multiple Gaussian. CD_2(s), CD_2(ot) and CD_2(as)

modes are assigned according to earlier report. [9] **(G)-(I)** Normalized SRS spectra of glucose isotopologues and starch they labeled. The similar Raman peaks between glucose isotopologues and starch they labeled showing direct condensation from glucose to starch. The spectra of glucose isotopologues-labeled starch were collected in situ. **(J)-(L)** Normalized SRS spectra of glucose isotopologues-D_2O labeled starch and fitted by multiple Gaussian. Peaks at around 2170 cm^{-1} is allocated to CD mode and some fermion resonance with overtone of CD_2 mode, 2120 cm^{-1} is allocated to CD_2 symmetric mode, around 2240 cm^{-1} is allocated to CD_2 anti-symmetric mode.

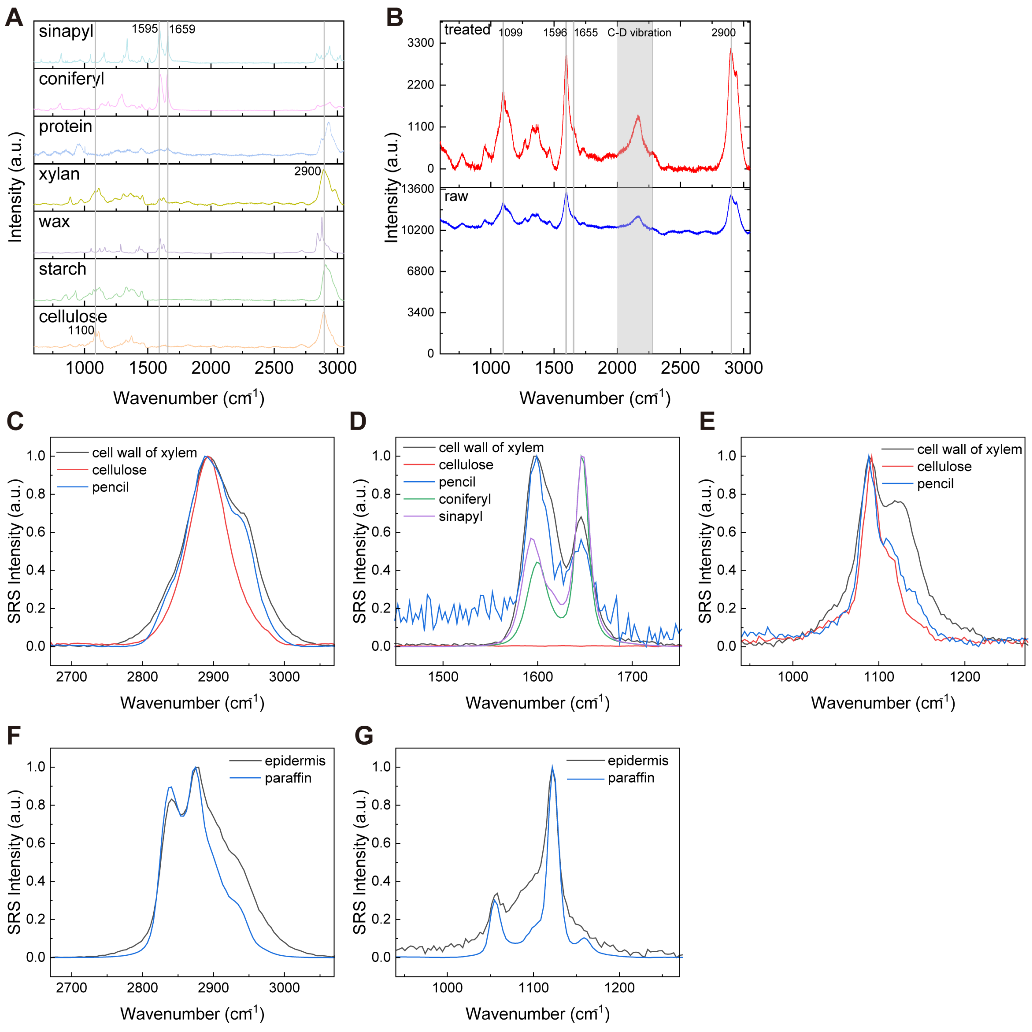

Figure S8. Raman spectra analysis of chemical constituents at various wavenumber range with standard samples. (A) Spontaneous Raman spectra of standard samples which are main components of plant cells. **(B)** Spontaneous Raman spectra of a stem cross-section of *A. thaliana* plants cultured with 50% D_2O/H_2O. Treated with auto-fluorescence background subtraction and denoising using 2rd-order polynomial with savitzky-golay filter. **(C)-(E)** Normalized SRS spectra of in situ cell wall of xylem and its possible standard samples and wood shavings from pencil. Spectra of cellulose are consistent with which of cell wall of xylem around frequencies at 2900 cm^{-1} (**C**) and 1100 cm^{-1} (**E**). Coniferyl and sinapyl are composition of lignin and have similar Raman shifts with in situ cell wall of xylem at 1600 cm^{-1} and 1650 cm^{-1}. So that xylem is mainly composed of cellulose and lignin [11]. The spectra of wood shavings rich in lignocellulose and xylem are almost the same at any frequency intervals, showing almost the same composition. **(F)(G)** Normalized SRS spectra of in situ wax around

epidermis and its possible standard sample paraffin. Both of them show Raman peaks around 2900 cm^{-1} and 1100 cm^{-1}, therefore constituent of in situ wax around epidermis is clear.

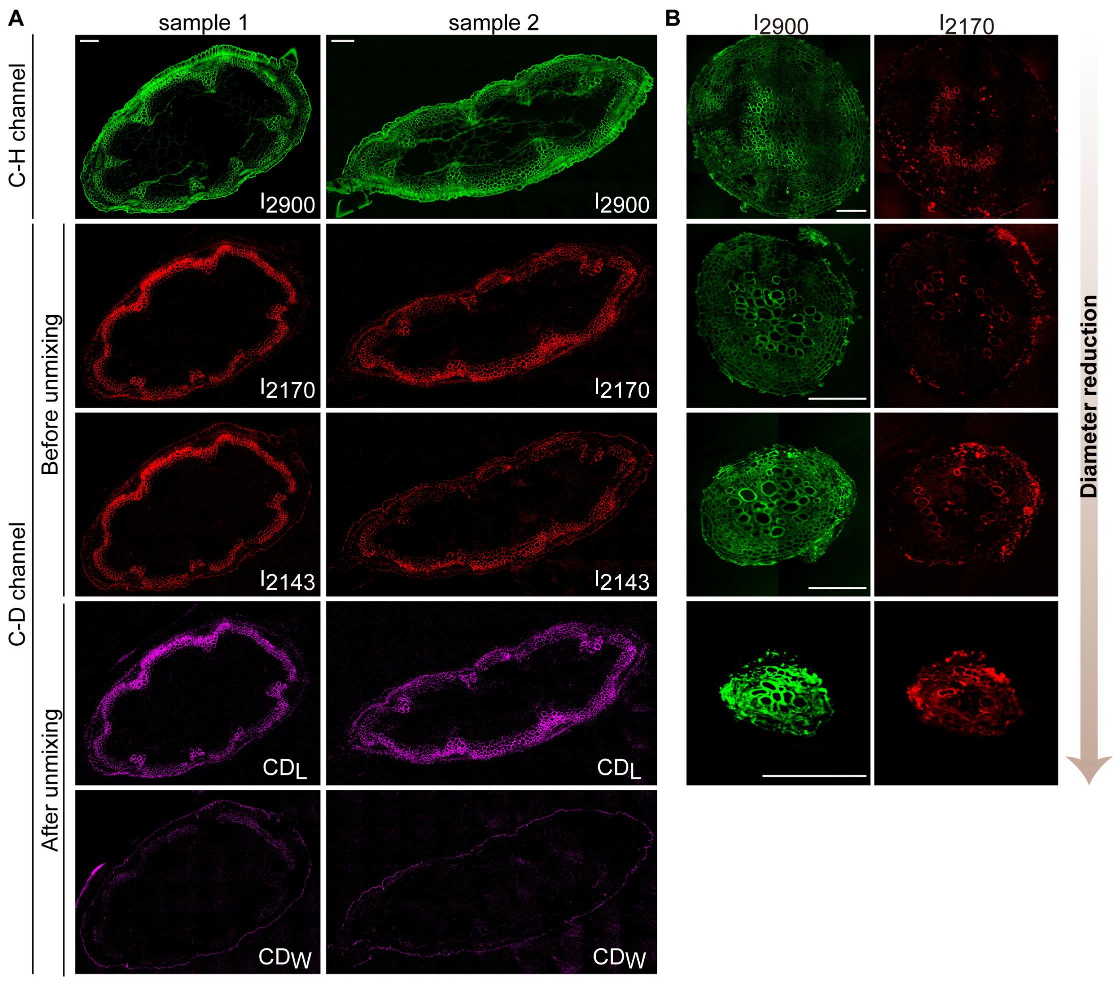

Figure S9. SRS images of stem and root cross-sections. (A) SRS images of two stem cross-sections at Raman frequencies of C-H stretch (2900 cm^{-1}, green); C-D stretch (2170 cm^{-1} and 2143 cm^{-1}) before spectral decomposition (red), after spectral decomposition (magenta) into lignocellulose (CD_L) and wax (CD_W). **(B)** SRS images of the cross-sections taken with varying diameters of hypocotyl/root with C-H band (2900 cm^{-1}, green) and C-D band (2170 cm^{-1}, red). Scale bars, 100 μm.

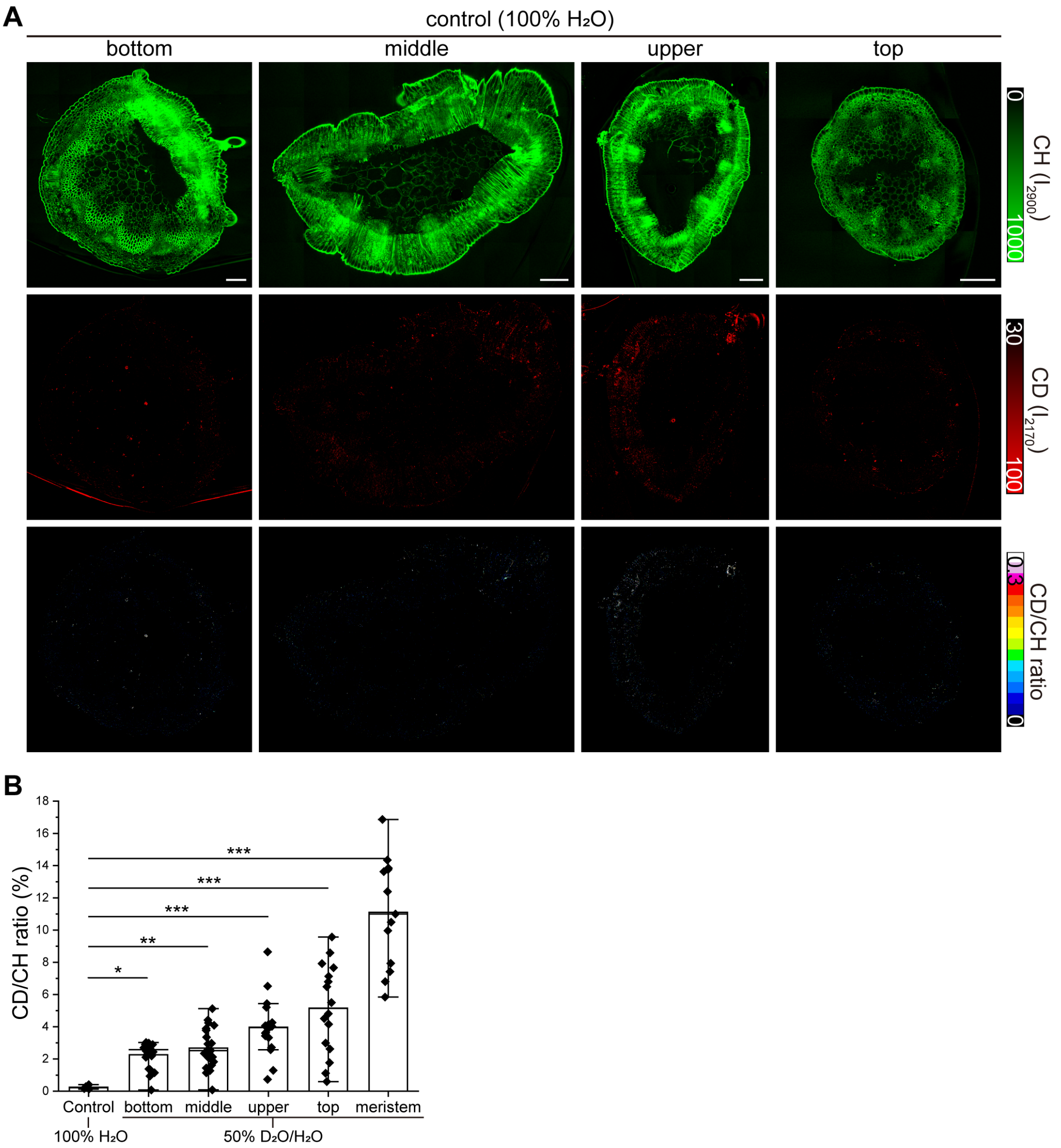

Figure S10. SRS images and quantifications of stem cross-sections of *A. thaliana* with 100%H_2O cultured. (A) SRS images of stem cross-sections at Raman frequencies of C-H stretch (2900 cm^{-1}, green), C-D stretch (2170 cm^{-1}, red) and ratiometric images of CD/CH before spectral decomposition along the axial direction of the pure H_2O cultured plant, from the bottom to the tip. Pure H_2O cultured plants of *A. thaliana* showed negligible C-D signal. Scale bars, 100 μm. **(B)** Quantification of the mean ratio intensities (means ± SDs) of the transverse cross-sections between H_2O cultured and 50%D_2O cultured plants. ***, $p<0.001$; **, $p<0.01$; *, $p<0.1$; ns, $p>0.05$ in ANOVA test. n = 4 for control (100% H_2O cultured).

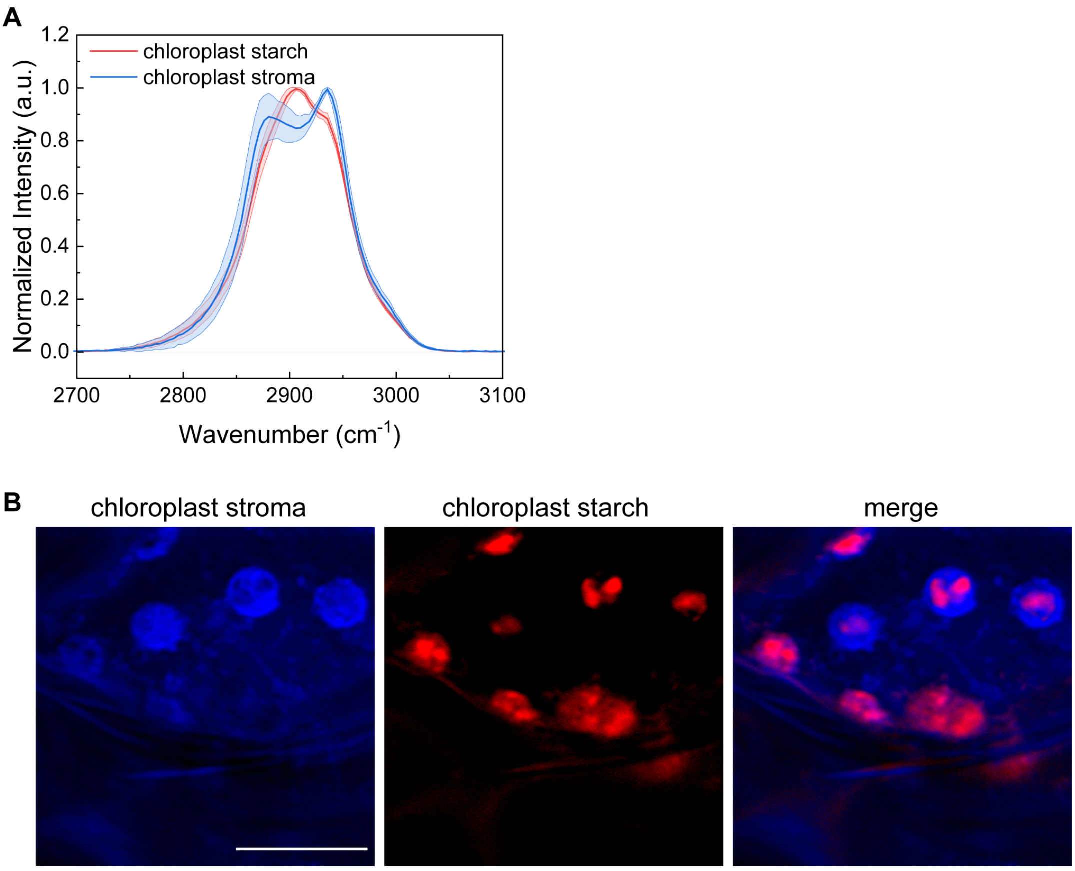

Figure S11. SRS microscopy unveils internal structure of chloroplast after chlorophyll clearing. (A) SRS spectra of chloroplast starch and chloroplast stroma at C-H region collected from various leaves of *A. thaliana* after chlorophyll clearing with ethanol. **(B)** MCR reconstructed concentration maps of chloroplast stroma (blue), chloroplast starch (red) and their overlay merged images of a sample of chlorophyll-cleared leaf tissue of *A. thaliana*. Scale bar, 10 μm.

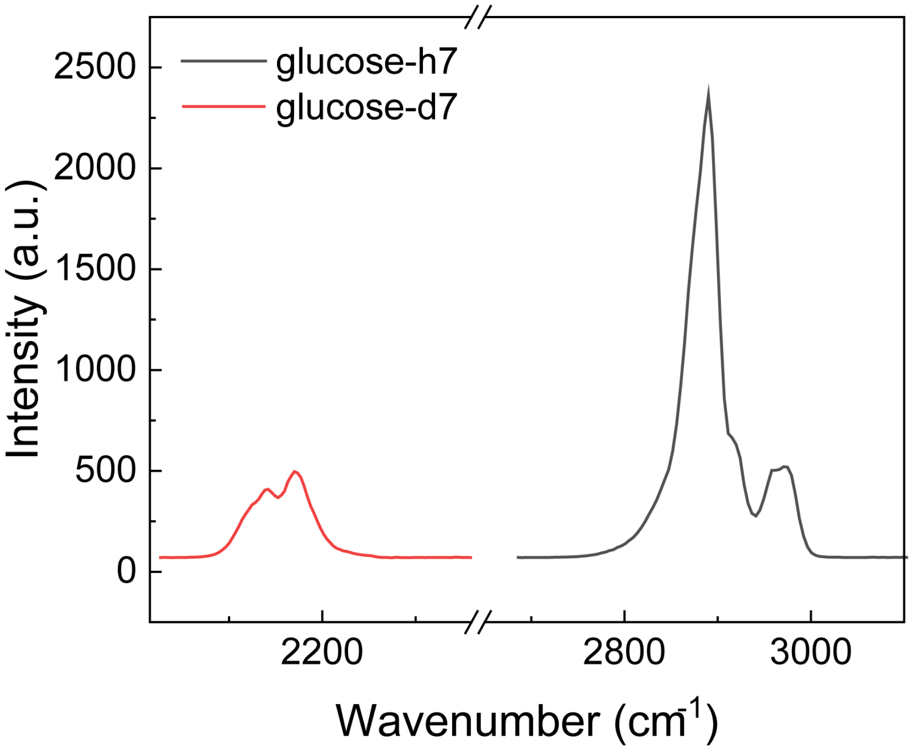

Figure S12. SRS spectra of crystalline D-glucose (black) and D-glucose-d7-1,2,3,4,5,6,6 (red). The ratio of integral over the spectral area between the cell-silent spectra region and high wavenumber region can be calculated as:

$$\frac{CD}{CH} = \frac{54983.28325}{157801.6755} = 0.348 \qquad (1)$$

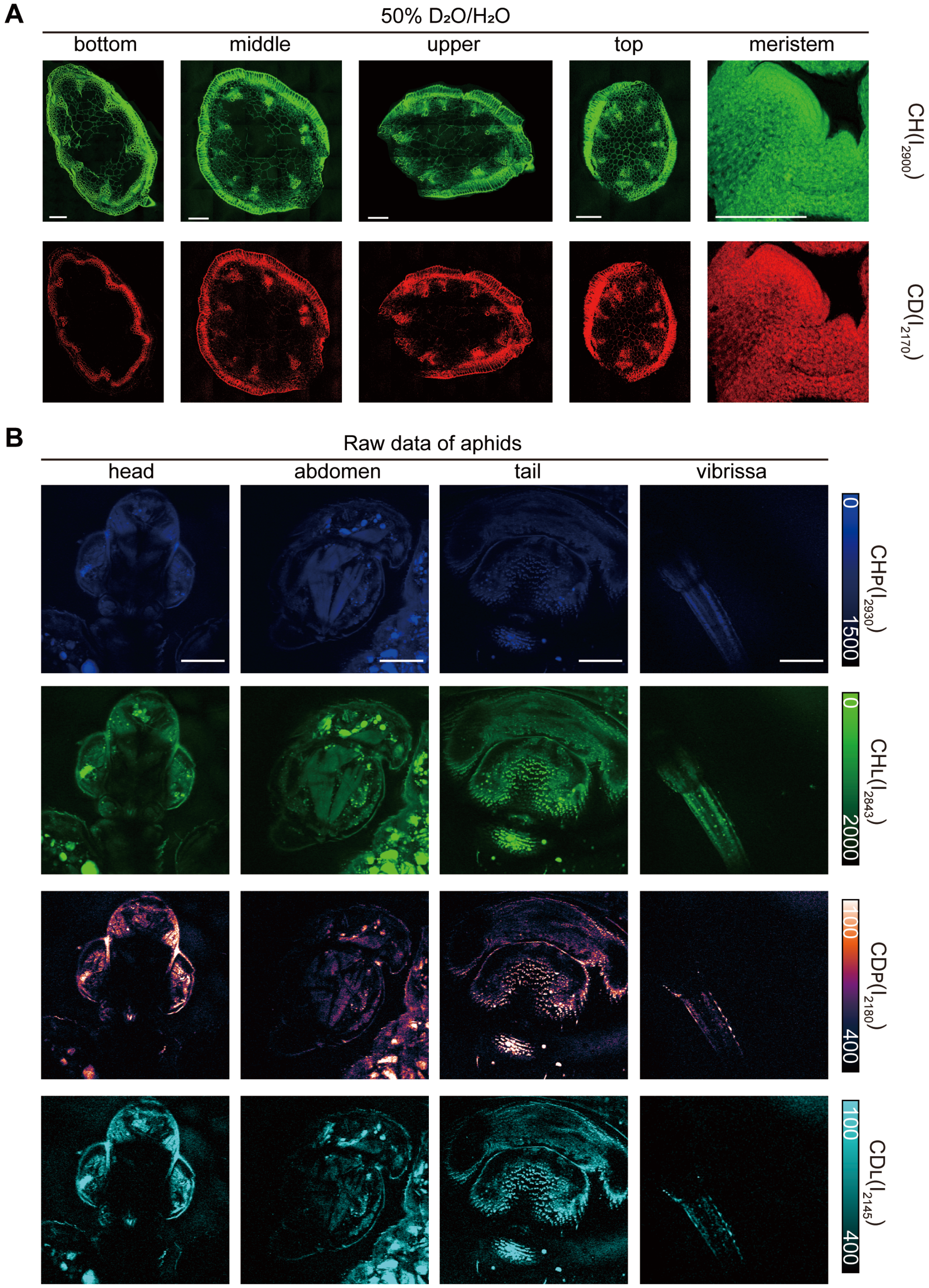

Figure S13. The SRS image before spectral unmixing. (A) The SRS image acquired for Figure 4D before spectral unmixing. Scale bars, 100 μm. **(B)** The SRS image acquired for Figure 6D before spectral unmixing. Scale bars, 50 μm.

Supplementary Table 1. Analytical methods for metabolism detection.

Method	Advantages	Disadvantages	Spatial resolution	Temporal resolution
Mass spectrometry (MS)	High sensitivity; High accuracy; High-throughput detection and quantification	No spatial information; Compounds with the same mass-charge ratio cannot be separated; Complex sample preparation	-	-
MS imaging (MSI)	High sensitivity; Localization of metabolite distribution in tissues	Complex sample preparation (need vacuum-dried and matrix coating); Limited in its ability to analyze water-rich materials	1.8 [1] - 200 [2] μm	5-15 minutes [1]
Nuclear Magnetic Resonance (NMR)	Precise quantification and reproducibility; Large field and deep imaging	Expensive cost of instrument; Large amount of samples; Insufficient spatial resolution;	100 μm [3] - sub-millimeter [4]	minutes [6]
Positron emission tomography (PET)	High sensitivity; Non-invasive; In-vivo; A complete 3D image	Radioactive; Insufficient spatial resolution; Only short-time metabolism can be detected	~ 1 mm [7]	minutes - hours [8]
Micro autoradiography	High sensitivity; High spatial resolution	Radioactive; Complex sample preparation; Limited to elements with suitable half-life	0.5 ~ 2 μm [10]	~ 5 days [12]
Fluorescence microscopy	High sensitivity; High spatial resolution; Easy sample preparation	Bulky fluorophores	200-300 nm [13]	Reach to 30 ms [14]
Spontaneous Raman (SR) spectroscopy & microscopy	Non-invasive; Easy sample preparation; Full spectral information	Insufficient spatial and temporal resolution	~ 0.5 μm [15]	~ 10 minutes [15]
Stimulated Raman scattering (SRS) microscopy	Non-invasive; High sensitivity; High spatial resolution; Easy sample preparation	Limited to parasitic background (transient absorption)	~ 350 nm	~ 1 s

Supplementary Table 2. Characteristic Raman peaks and states of chemical species.

Molecule	Spectral region	Raman Band (cm^{-1})	Measured state (in vitro/in vivo)	Localization in text
Starch	C-H stretching vibration	2908-2910	In vivo (root tip of radicle, seed), in vitro (standard sample)	Fig.2A, Fig.5B, Fig.S3
Protein		2930-2938	In vivo (root tip of radicle, SAM, aphids), in vitro (standard sample)	Fig.2A, Fig.4B, Fig.6A, Fig.S3
Lipid droplets		2845-2850	In vivo (root tip of radicle, seeds, aphids), in vitro (standard sample)	Fig2A, Fig5B, Fig.6A, Fig.S3
Cellulose (cell wall/vascular tissue)		2896-2900	In vivo (root tip of radicle, stem), in vitro (standard sample)	Fig.2A, Fig.3B, Fig.S3, Fig.S7C
Wax		2840-2843, 2874-2880	In vivo (stem, aphids), in vitro (standard sample)	Fig.3B, Fig. 6A, Fig.S7F
Glucose-d1-1	C-D stretching vibration	2156-2210	In vitro (Standard sample)	Fig.S6G
Glucose-d2-6,6		2122-2123, 2180-2189, 2240-2245		Fig.2F, Fig.S6H
Glucose-d7-1,2,3,4,5,6,6		2123-2130, 2160-2170, 2220-2230		Fig.2F, Fig.S6I
Glucose-d1-labeled starch		2186	In vivo (root tip of radicle)	Fig.S6G
Glucose-d2-labeled starch		2121, 2186		Fig.S6H
Glucose-d7-labeled starch		2135, 2177		Fig.S6I
D2O+Glucose-d1-labeled starch		2170	In vivo (root tip of radicle)	Fig.S6J
D2O+ Glucose-d2-labeled starch		2122, 2168, 2180, 2245	In vivo (root tip of radicle, seed)	Fig.S6K
D2O+ Glucose-d7-labeled starch		2119, 2170, 2235		Fig.S6L
D_2O-labeled starch		2170		Fig.2D, Fig.2F, Fig.5B
D_2O-labeled protein		2180	In vivo (SAM, aphids)	Fig.4B, Fig.6B
D_2O-labeled lipid		2147	In vivo (seed, aphids)	Fig.5B, Fig.6B
D_2O-labeled lignocellulose		2170	In vivo (stem)	Fig.3B
D_2O-labeled wax		2143	In vivo (stem, aphids)	Fig.3B, Fig.6B

References

[1] M. Iijima, T. Yoshida, T. Kato, M. Kawasaki, T. Watanabe, S. Somasundaram, *J. Exp. Bot.* **2011**, *62*, 2179.

[2] R. Shroff, F. Vergara, A. Muck, A. Svatoš, J. Gershenzon, *Proc. Natl. Acad. Sci.* **2008**, *105*, 6196.

[3] C. W. Windt, F. J. Vergeldt, P. A. De Jager, H. Van As, *Plant Cell Environ.* **2006**, *29*, 1715.

[4] P. T. Toi, H. J. Jang, K. Min, S.-P. Kim, S.-K. Lee, J. Lee, J. Kwag, J.-Y. Park, *Science* **2022**, *378*, 160.

[5] a) D. V. L. Mohamed Mathlouthi, *Carbohydr. Res.* **1980**, *81*, 203; b) V. S. Gorelik, L. P. Avakyants, A. V. Skrabatun, *J. Raman Spectrosc.* **2022**, *53*, 128.

[6] M. Gussoni, F. Greco, A. Vezzoli, T. Osuga, L. Zetta, *Magn. Reson. Imaging* **2001**, *19*, 1311.

[7] M. Hubeau, K. Steppe, *Trends Plant Sci.* **2015**, *20*, 676.

[8] A. Hahn, M. B. Reed, C. Vraka, G. M. Godbersen, S. Klug, A. Komorowski, P. Falb, L. Nics, T. Traub-Weidinger, M. Hacker, R. Lanzenberger, *Eur. J. Nucl. Med. Mol. Imaging* **2023**, *51*, 1310.

[9] G. Z. Giovanna Longhi, Germana Paterlini, Laure Ricard, Sergio Abbate, *Carbohydr. Res.* **1987**, *161*, 1.

[10] N. Musat, R. Foster, T. Vagner, B. Adam, M. M. M. Kuypers, *FEMS Microbiol. Rev.* **2012**, *36*, 486.

[11] a) N. Zhu, Y. Yang, M. Ji, D. Wu, K. Chen, *Hortic. Res.* **2019**, *6*, 72; b) Y. Zeng, J. M. Yarbrough, A. Mittal, M. P. Tucker, T. B. Vinzant, S. R. Decker, M. E. Himmel, *Biotechnol. Biofuels* **2016**, *9*, 256.

[12] L. Hu, H. Sun, R. Li, L. Zhang, S. Wang, X. Sui, Z. Zhang, *Plant Cell Environ.* **2011**, *34*, 1835.

[13] R. Decou, H. Serk, D. Ménard, E. Pesquet, *Methods Mol. Biol.* **2017**, *1544*, 233.

[14] P. P. Mondal, *Front. Mol. Biosci.* **2014**, *1*.

[15] M. Kamp, J. Surmacki, M. Segarra Mondejar, T. Young, K. Chrabaszcz, F. Joud, V. Zecchini, A. Speed, C. Frezza, S. E. Bohndiek, *Nat. Commun.* **2024**, *15*.
